# Ionization and Chain Size of Weak Polyelectrolytes in Semidilute Regime

**DOI:** 10.1021/acs.macromol.5c00895

**Published:** 2025-09-08

**Authors:** Lucie Nová, Miroslav Štěpánek, Iryna Morozova, Zdeněk Tošner, Filip Uhlík

**Affiliations:** Department of Physical and Macromolecular Chemistry, Faculty of Science, 112302 Charles University, Hlavova 8, 128 00 Praha, Czech Republic

## Abstract

We present the relations between chain size, correlation length, and degree of ionization for linear weak polyelectrolytes in the semidilute regime. In the semidilute regime, SAXS provides the correlation length(s), ξ, but cannot provide the chain size characteristics. Similarly, pH measurements and the Henderson–Hasselbalch equation do not yield a realistic estimation of the degree of ionization of weak polyelectrolytes in the semidilute regime. On the other hand, simulations provide all these: end-to-end distance *R*_ee_, radius of gyration *R*_g_, degree of ionization α. We measured SAXS profiles of poly­(acrylic acid) at various degrees of neutralization in the semidilute regime. We compared the experimental scattering curves with their counterparts from coarse-grained Hamiltonian Monte Carlo simulations in terms of the position of the polyelectrolyte peak, q*, and the fractal dimension, *d*_f_. Regarding q*, our results not only are in mutual agreement but also obey the previously predicted scaling relations in the limit of high α values. In particular, we obtained *R*_ee_ ∝ ξ^0.3^ for α >  0.3, *R*_ee_ ∝ α ^4/7^ for 0.1 < α < 0.5, *R*_ee_ ∝ α^1/7^ for α  > 0.5 and *R*_ee_ approximately constant for very low values of α.

## Graphical abstract

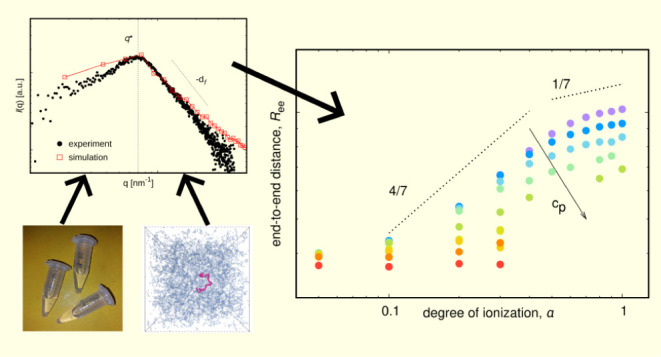

## Introduction

Polyelectrolytes are polymers that carry ionizable groups. *Strong* polyelectrolytes in solutions are fully ionized, but the degree of ionization of *weak* polyelectrolytes depends on external conditions such as pH, ionic strength, temperature, and polymer concentration. The degree of ionization of weak polyelectrolytes at a given pH is lower than the degree of ionization of the respective short oligoelectrolytes or monomeric electrolytes.^1–4^ This result is usually presented as titration curves; the titration curve of a polyelectrolyte lies below the “ideal” titration curve of a monomeric electrolyte.^5–7^ This is often taken for granted, but we should not forget that these results hold for low concentrations, far below the *overlap concentration*.

The situation above the overlap concentration, in the semidilute regime, where the polymer coils are interpenetrated, is different.^8^ The ionization of neighboring ionizable units depends not only on the conformation of a single chain but also on the conformations of neighboring chains. The ionization is further suppressed by the spatial constraints caused by excluded volume. Moreover, counterion condensation,^9–11^ which is often used to explain the conformation-ionization interplay of chains at low concentrations,^6,7^ has limited possibilities to contribute to the conformation-ionization states of individual chains at high concentrations. While the titration behavior of weak polyelectrolytes below the overlap concentration has been extensively studied (reviewed, e.g., in refs 12–14 ), we know actually very little about the ionization behavior of weak polyelectrolytes in the semidilute regime.^15^ Although some experimental studies have already addressed this issue, theoretical description and, therefore, true understanding of the problem lies behind. The valid concepts developed and used for neutral polymers cannot be “simply applied” to polyelectrolytes.^16^

The properties of polyelectrolyte systems, and the chain size within these systems, are usually explained in terms of scaling relations.^8,17,18^ The size of a neutral polymer chain is independent of the concentration below the overlap concentration and in the semidilute regime it is proportional to *c* ^–0.12^. The description of the size of a charged polymer chain is much more complicated.^16,17,19,20^ The most intriguing difference from a neutral chain is the occurrence of many more regimes. Both the scaling coefficients and the concentrations that divide individual regimes strongly depend on the effective charge on the chain^16,17,19^ and on the presence of monovalent^21,22^ and multivalent counterions.^21,23,24^ These scaling relations, valid for *strong* polyelectrolytes, have been confirmed experimentally, using rheology and scattering techniques (see, e.g. refs 21, 22, 25, and 26). Besides this, computer simulations can predict these relations with quantitative accuracy.^27,28^ Note that the effective charge on the chain in the case of *weak* polyelectrolytes depends not only on the presence of salt ions, but also on pH, and very importantly, due to electrostatic interactions, on spatial constraints generated by the chain overlap and entanglement. Unfortunately, the scaling relations for semidilute and concentrated solutions of *weak* polyelectrolytes have not been developed yet,^29^ and there are no counterpart data from other methods, like computer simulation or scattering experiments to validate them.

In this study we use molecular simulations and small-angle X-ray scattering to investigate the ionization and conformation behavior of *weak* polyelectrolyte systems in the semidilute regime.

## Methods

### Molecular Simulations

#### Coarse-Grained Model

We used a coarse-grained model,^6,30,31^ which represents monomer units, counterions, and co-ions as soft spheres interacting via bonding (eq 1), nonbonding (eq 2), and Coulomb potentials. All explicit particles are characterized by their diameters, repulsion and attraction parameters, and reactivity, while the solvent is an implicit, structureless continuum characterized by its dielectric permittivity. Periodic boundary conditions and a cubic unit cell are used.

The bonded beads are connected via a harmonic potential

1 $$U_{b}=\varepsilon_{b}{\left(r-r_{0}\right)}^{2}$$

where *ε*_b_ = 80 *k*_B_ *T* is the strength of the massless spring, *r* is the distance between two adjacent beads, and *r*_0_ = 0.30 nm is the distance at the minimum of *U*_b_. The bead size 0.30 nm roughly corresponds to the size of two C–C bonds of a vinyl-type polymer.

The excluded volume is described via a soft short-range repulsion potential^6,32^

2 $$U_{\mathrm{sr}}=\varepsilon_{\mathrm{sr}}\frac{{\left(r-r_{c}\right)}^{2}}{r^{2}},\mathrm{for}\;r<r_{c}$$

and zero otherwise, where *ε*_sr_ = 1.0 *k*_B_ *T* and *r*_c_ = *r*_0_ is the cutoff. This potential is smooth at the cutoff.

To simplify our coarse-grained model, we employed the same excluded volume potential form with the same *ε*_sr_ for all explicit particles. The excluded volume parameter in the pair interaction, *r*_c_, is approximated as the arithmetic mean of the respective radii of the interacting particles $r_{c}=\frac{r_{1}+r_{2}}{2}$ . The polymer bead excluded volume radius is set to 0.3 nm. The excluded volume radii of the ions (H^+^, OH^–^) are 0.1 nm, which roughly corresponds to the van der Waals radius of the solvated hydrogen ion.

Long-range electrostatic interactions were calculated using the Ewald summation.^33^ We used either partially charged chains with fixed ionization degree to model *strong* polyelectrolytes, or we dynamically varied the ionization of the polyelectrolyte chains within the reaction ensemble^30,31,34^ to model *weak* polyelectrolytes (see Reaction Model for more details).

The number of polymer particles was kept 10^4^ in all simulations; the system contained 200 chains with 50 beads. The chain length is a reasonable compromise; these chains already adhere polyelectrolyte behavior,^6^ but are still short enough to keep reasonable simulation costs. We simulated these systems at various polymer concentration values.

#### Reaction Model

The monomer beads in the *strong* polyelectrolyte model do not undergo the ionization reaction and their charges are fixed throughout the simulation. The presence of negatively charged beads is compensated for by the appropriate number of oppositely charged counterions H^+^.

On the contrary, all monomer beads in the *weak* polyelectrolyte model can undergo dynamic ionization as weak acids:^6^

3 $$\mathrm{HA}⇄A^{-}+H^{+}$$

We mimic various pH conditions by means of systematic variation of p*K*_A_.^13,14^ The ionization of the PAA polymer beads varies dynamically with the reaction constant, which is the input of the simulation. When the PAA bead undergoes the reaction in the forward direction, we switch the PAA monomer charge from 0 to −1, and compensate for the presence of the charged polymer bead by inserting an oppositely charged counterion, H^+^. Similarly, when applying the reverse reaction, we switch the charge of the ionized polymer bead from −1 to 0 and delete the randomly chosen H^+^ ion.

In this work, we either set fixed p*K*_A_ and measured the charge on the polymer chains as described above or, we set fixed charge on the polymer chains and we employed the “shuffling reaction”:

4 $$\mathrm{HA}+A^{-}+H^{+}⇄A^{-}+\mathrm{HA}+H^{+}$$

Using this reaction, the positions of charged monomers in the systems can rearrange and mimic the behavior of *weak* polyelectrolytes, while keeping constant the overall degree of ionization for straightforward comparison with *strong* polyelectrolytes.

Apart from the ionization reaction, we employed the water autoprotolysis reaction:

5 $$H_{2}O⇄H^{+}+{\mathrm{OH}}^{-}$$

The reaction in eq 5 took part in the simulations of both *weak* and *strong* polyelectrolyte systems. It mimics the exchange of H^+^ and OH^–^ ions between the system and a reservoir.^13,14^ The pH value (eq 6) was obtained from the H^+^ concentration in the simulation box:

6 $$\mathrm{pH}=-\log\frac{N\left(H^{+}\right)}{N_{A}V_{\mathrm{box}}}$$

#### Permittivity

Electrostatic interactions are long-range and their strength is determined by the relative permittivity of the medium ϵ_m_. Because our system comprise large portions of polymeric chains, the permittivity of the medium cannot be simply approximated by the permittivity of water, ϵ_w_. We calculated the permittivity of the medium according to the Maxwell–Garnett formula^35^

7 $$\frac{{\text{ϵ}}_{m}-{\text{ϵ}}_{p}}{{\text{ϵ}}_{m}+2{\text{ϵ}}_{p}}=\Phi_{w}\frac{{\text{ϵ}}_{w}-{\text{ϵ}}_{p}}{{\text{ϵ}}_{w}+2{\text{ϵ}}_{p}}$$

where ϵ_p_ is the relative permittivity of the polymer in the dry state and Φ_w_ is the volume fraction of water. The particular values of Φ_w_ and ϵ_m_ are listed in Table S2.

In this work, we have ϵ_w_ = 80 and ϵ_p_ = 5 (common values for water and nonconducting polymers, respectively). Φ_w_ varies with the system density and is calculated assuming that explicit polymer particles are spheres of diameter *r*_0_.

#### Simulation Protocol

We used our in-house implementation of the Hamiltonian Monte Carlo model in the reaction ensemble.^6,30,31^ Using this method, we study simultaneously both the conformational and ionization behavior. The simulation proceeds as follows. First, we randomly choose between the conformational and reaction step.

In the reaction step, we randomly choose one of the employed reactions, and then choose between the forward and backward directions of this reaction. In terms of the ionization reaction (eq 3 or eq 4), this means that we chose between performing a dissociation or an association reaction. Taking into account the above, we randomly chose reactants and/or randomly insert products of the reaction. Subsequently, we submitted the proposed state to the Monte Carlo (MC) procedure, either accepting or rejecting the proposed state.^13,31,34^

In the conformational step, we perform a time evolution using a Molecular Dynamics (MD) scheme followed by the standard MC procedure, accepting or rejecting the proposed state using the Metropolis criterion.^36^ In this study, we used 50 MD steps to prepare the MC proposal. The MD time step length was *d*_*t*_ = 0.05 time simulation units. In the randomly chosen MD time steps, we randomly decreased the MD time step length to improve the MC acceptance ratio.

The simulations were performed at 300 K. The size of the bead was 0.30 nm, and its mass was 50 atomic mass units.

The entire simulations usually comprise >10^3^ τ_int_ in *R*_g_, which is the slowest evolving quantity. τ_int_ is the integrated autocorrelation time obtained from the Wolff autocorrelation estimation method.^37^

We used our own programs and scripts for the postsimulation data treatment.

### Materials

Poly­(acrylic acid), *M*_*w*_ = 240 kg/mol, 25% (w/w) aqueous solution, was purchased from Sigma-Aldrich. The samples for SAXS measurements were obtained by appropriate dilution of the 25% stock solution. pH was adjusted by adding sodium hydroxide (LachNer, Czech Republic).

### Small Angle X-ray Scattering (SAXS)

SAXS experiments were performed at the BIOCEV center (Vestec, Czech Republic) with a SAXSpoint 2.0 instrument (Anton Paar, Austria) equipped with an Excillum MetalJet C2+ X-ray source and with an Eiger R 1M detector (Dectris, Switzerland). The active area of the detector and the pixel size were 79.9 mm × 77.2 mm and 75 μm × 75 μm, respectively. The wavelength of the X-ray source was λ = 0.1348 nm. Measurements were conducted at 20 °C in a silicon nitride cell at the sample-to-detector distance of 0.82 m, corresponding to the *q* vector range from 0.04 to 4 nm^–1^. SAXS curves were obtained by azimuthal averaging of scattering patterns for the sample and the solvent and subtracting the solvent intensities from those for the samples. The temperature was controlled using a TCStage 150 temperature controller. The raw scattering data sets are available in Zenodo (10.5281/zenodo.15553098).

### Nuclear Magnetic Resonance (NMR)

Inserts with D_2_O and traces of DSS were used for field-frequency lock and chemical shift referencing. ^1^H and ^13^C NMR spectra were recorded using a Bruker Avance III spectrometer operating at proton Larmor frequency of 600 MHz equipped with a standard BBO probe. Chemical shift values for evaluating ionization state were obtained as center of mass of the carbonyl region. Measurements of translational diffusion coefficients were performed using DSTEBP pulse sequence^38^ with 32 steps in gradient strength ranging from 5% to 98% of the maximal value of 50 G/cm. The gradient pulses of 2.5 ms duration were separated by 1 s diffusion delay. The absolute diffusion coefficients were calibrated assuming the HDO diffusion coefficient equal to 1.9 × 10^–9^ m^2^ s^–1^ at 25 °C.^39^ Data were processed using the spectrometer software (Topspin 3.6, Bruker) and the intensity decays of the DOSY data were fitted using self-written scripts in gnuplot.

## Results and Discussion

### SAXS

#### Scattering Curves

The raw simulation data provide coordinates of all particles involved in the simulation drawn from the canonical probability distribution, which allows us to calculate the corresponding scattering function

8 $$I\left(q\right)=\frac{1}{n}\underset{ij}{\sum }\left\langle \exp\left(-iq\cdot \left(r_{i}-r_{j}\right)\right)\right\rangle$$

where *q* is the scattering vector and *r*_*i*_ and *r*_*j*_ are the positions of individual particles in the sample. As the simulations use periodic boundary conditions, we calculated the scattering functions in eq 8 only for selected values of *q* = 2π*k*/*L*, where *L* is the size of a cubic simulation box and $k\in Z_{+}$ and averaged over different lattice directions with same length.

We subjected both experimental and simulation scattering curves to the same treatment and obtained from them two most intriguing characteristics: q* and *d*_f_ (Figure 1). More sample experimental SAXS curves are in Figures S18 and S19. The q* value (position of the “polyelectrolyte peak”) was calculated by fitting a second-order polynomial to the relevant section of the scattering curve near q*. When necessary, a previous spline smoothing was applied. The −*d*_f_ value is the slope of the part of the scattering curve beyond q* in the double logarithmic representation, and was obtained by least-squares fitting of the appropriate part of scattering curve to a power law (respectively, to a linear function in the double logarithmic representation). The *d*_f_ value can be interpreted as fractal dimension of the polymer coil; *d*_f_ = 1 for the fully extended conformation vs *d*_f_ = 2 for the Gaussian coil, and can be used as a measure of the curvature of the chain. Theory predicts

9 $$d_{f}=\frac{1}{\nu }$$

where ν is the scaling exponent *R*_ee_ ∝ *N* ^ν^.^40^

**Figure 1:**
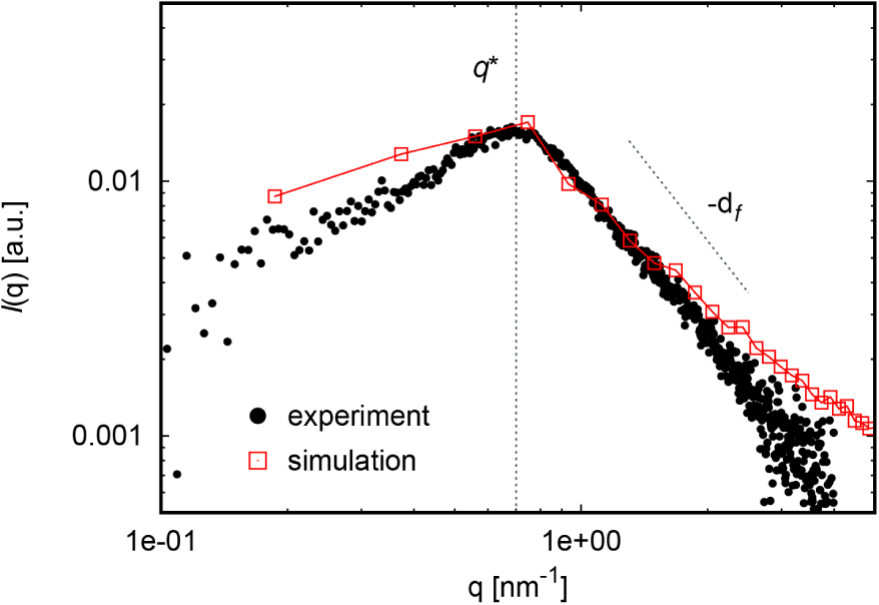
1:1 comparison of SAXS profiles from experiment and simulation at c/c* ≐ 1 and α = 0.4.

In some cases, like the most and the least concentrated experimental samples with very low and high α values, the q* and d_*f*_ values were statistically undetermined with too high uncertainty and were excluded from figures and discussion.

#### Comparison

We compared the dependence of q* and *d*_f_ on the polymer concentration (Figure 2 and Figure S2) and on its degree of ionization, α (Figure 3 and Figure S3). In both Figure 2 and Figure 3 we see a good agreement between experiment (full symbols) and simulation (empty symbols). The black crosses in this section (Figure 3) or in the corresponding Figures S1 and S3 come from Figure 6 and 8 in ref^25^ and Table 1 in ref 41.

**Figure 2:**
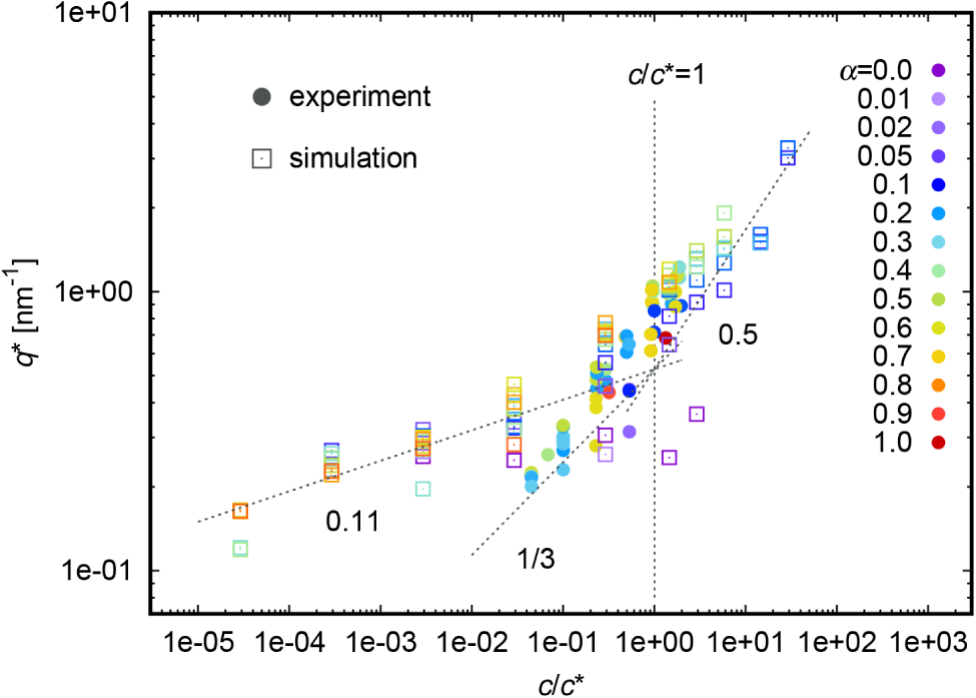
Variation of q* with PAA concentration.

**Figure 3:**
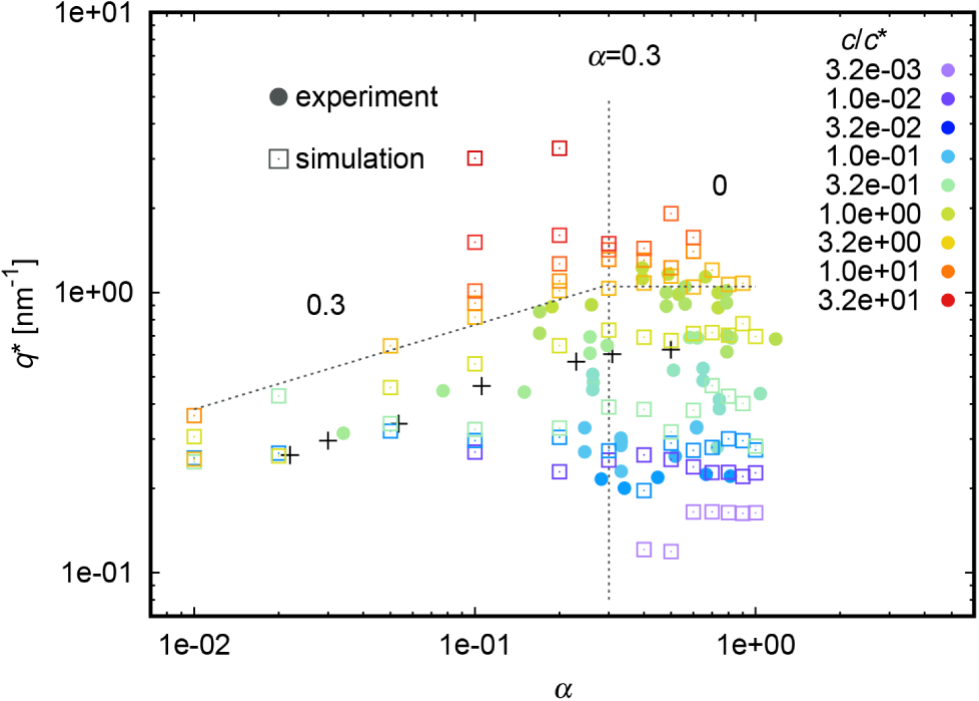
Variation of q* with α.

In both Figure 2 and Figure S2, we see two distinct regions, which could correspond to two distinct regimes: *c* < c* and *c* > c*. The overlap concentration differs for the experimental and simulation setups. For simulation, we estimate it from Figure S1 as a crossover of the two regimes; c* = 0.3 mol/L. We estimated the overlap (or rather entanglement^42^) concentration of our non-neutralized experimental sample as c* = 0.45 mol/L, using dynamic light scattering (for more details see Figure S13 and Figure S14) and DOSY NMR (for more details, see Figures S15–S17). We use the value c* = 0.45 mol/L for all experimental data in Figure 2 and all relevant figures. All concentration values have the meaning of amount of ionizable polyelectrolyte units per liter. In all figures, we use only one c* value for experimental samples and one c* value for simulation data (although we are fully aware that c* depends on the charge on the chain). This approach also enables an easier observation of the effects of the degree of ionization.

For *c* < c*, q* is the signature of a soft position order (“liquid-like order”) between the centers of mass of the chains, and for strong polyelectrolytes, it scales as q* ∝ *c* ^1/3^,^25^ which corresponds to the interchain correlations in dilute solutions.^43^ Our experimental data in this regime are scarce, because experiment targeted only the semidilute regime. In this case, the depicted line with label 1/3 in Figure 2 does not come from a fitting procedure but serves just as a guide to the eye. However, we found the exponent 0.11 from our “simulation SAXS” curves and in this case the depicted scaling value comes from the least-squares fitting procedure. This concentration range is usually called the dilute regime, but we need to take into account that, for polyelectrolytes, this is below the overlap concentration c*, but above the interaction concentration *c*_int_.^17^ Polyelectrolyte chains, unlike neutral ones, interact with each other in dilute solutions as well because the electrostatic interactions are long-range.

In the semidilute regime, q* is related to the correlations between polyion segments and scales with concentration with exponent 0.5 (here obtained from a fitting procedure), which agrees with previously published results^24,25^ for strong polyelectrolytes. Moreover, Figure S1 shows agreement in direct comparison of our data with previously published data for sodium polystyrenesulfonate, NaPSS (strong polyelectrolyte), taken from Figure 6 in ref 25. This might raise the question whether there is any difference between weak and strong polyelectrolytes when it comes to the semidilute regime.

The color code in Figure 2 distinguishes various degrees of ionization. There is only a very tiny difference between the q* values for different degrees of ionization. Lower degrees of ionization correspond to slightly lower q*, but the scaling is the same. The only exceptions are very low degrees of ionization (α = 0.01), where the system tends to behave more like composed of neutral chains than of the charged ones. Note that strong polyelectrolyte chains are fully ionized in solution. Apparently, the charge on the chain does not affect the position of the “polyelectrolyte peak”, q* even for weak polyelectrolytes. The cluster of simulation q* points around *c*/c* = 1 suggests that the overlap concentration depends on the charge on the chain,^17^ but we lack data to be able to quantitatively confirm the previously published scaling relations.

The dependence of q* on the degree of ionization (Figure 3) also depicts the existence of two distinct regimes, commonly observed for strong polyelectrolytes;^25^ we observe the scaling exponents 0.3 and 0 below and above α = 0.3, respectively. Both exponents come from a least-squares fitting procedure. Note that theory predicts q* ∝ α^2/7^ = 0.29.^25^ The black crosses are data for sodium polystyrenesulfonate, NaPSS (strong polyelectrolyte) from Figure 8 in ref^25^. Depicted q* values are lower for lower concentrations.^18^ The color code in Figure 3 has the meaning of various concentrations. We need to point out that the above-mentioned behavior with two regimes holds for higher concentrations, in the semidilute regime. This behavior fades away with decreasing concentration. Finally, q* is independent of α in the dilute solution limit.

For better visualization of q* dependencies on concentration in Figure 2 and degree of ionization α in Figure 3, we plotted selected data together in Figure 4 for experiment and two types of simulations; grand-canonical with fixed pH – p*K*_A_ (using the ionization reaction shown in eq 3) and canonical with fixed degree of ionization (using the shuffling reaction shown in eq 4). The degrees of dissociation of individual chains fluctuate in both cases with others chains serving as a bath in the later case. Both types of simulations nicely agree. The agreement with somewhat scattered experimental points with larger experimental errors is also good.

**Figure 4:**
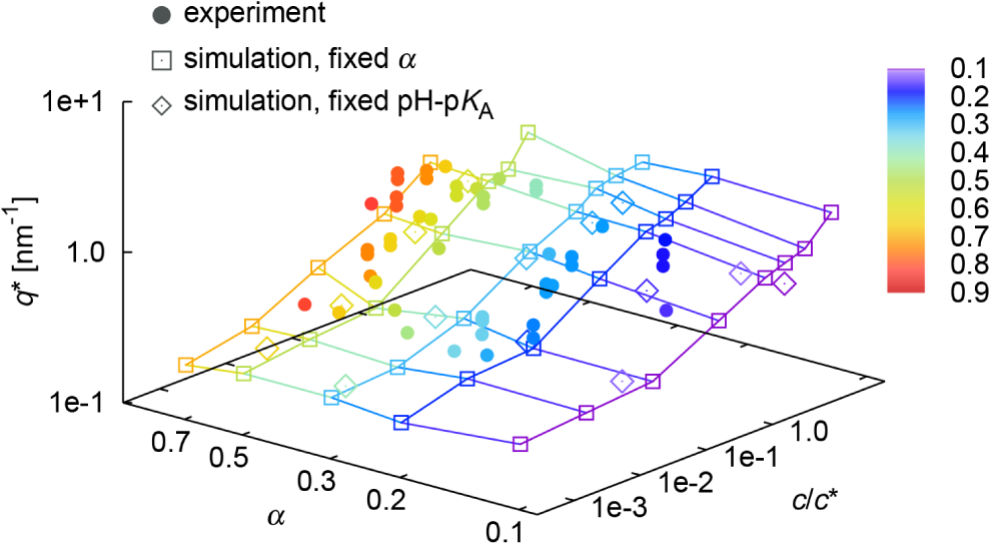
Variation of q* with PAA concentration and α.

The dependence of d_*f*_ values on the polymer concentration in Figure S2 differs for experimental and simulation data series, but despite this, we wanted to be honest and discuss it.^44^ For experimental data, we see uniform increase of d_*f*_ values with concentration; the values for lower degrees of ionization are lower, while the values for higher degrees of ionization are higher. For simulation data, we see a nonmonotonic dependence; slight increase below c* and then a decrease above c*. Unlike for experimental data, the *d*_f_ values are higher for lower degrees of ionization. It was shown^45^ that the *d*_f_ values from MC simulation of polyelectrolytes vary between the values 1 (rod) for higher electrostatic coupling and 1.5 for lower electrostatic coupling (compare *d*_f_ = 1.66 for self-avoiding walk) and that these values are size independent from chain length *N* > 32. Meanwhile, the *d*_f_ values from experiment values ∼2 correspond to a random walk (of polyelectrolyte blobs).^46^ The disagreement between the simulation and experimental dependencies of *d*_f_ in Figure S2 is caused by the fact that on shorter length scales, the used simulation model does not describe the structure of the chain realistically. The coarse-grained simulation model does not reflect the internal structure of the segments and therefore works reliably only at the length scales larger than the size of the polymer segment. SAXS or SANS measurements of fully ionized polyelectrolytes in dilute solutions provide the expected scaling regime with *d*_f_ = 1 only at lower *q*, while at *q* > 1 nm^–1^ the power law exponent already reflects the internal structure of the segments.^47–49^ Moreover, we used a model with implicit solvent that affects the value of *d*_f_.^28^

The dependence of *d*_f_ on the degree of ionization in Figure S3 is again different for experimental and simulation data series. While *d*_f_ increases uniformly with α for the experimental data, it decreases for the simulation data.

### Conformational Characterization

The scattering curves are usually used to obtain information about the internal structure and morphology of polymer systems. As mentioned above, in the case of polyelectrolytes, we can extract information about q* and relate it to the correlation length, ξ. Still, the meaning of the correlation length(s) in polyelectrolyte systems is not as straightforward as in the case of neutral chains.^16,24^ We cannot simply attribute the correlation length to the chain size, *R*. On the other hand, molecular simulations provide this information directly; we calculate the end-to-end distances (*R*_ee_) and radii of gyration (*R*_g_) from the *xyz* coordinates of individual polymer segments. We have shown in the previous paragraph that the q* values from simulations agree with the q* values from experiment and that we can trust the simulation data in terms of providing reliable structural data on polyelectrolytes. Now, it remains to show how the most significant polyelectrolyte feature in SAXS, q*, relates to the *R*_ee_ and *R*_g_ values.

#### Chain Size

Unlike in scattering experiments, simulations provide *xyz* coordinates of individual particles, which can be used for a straightforward evaluation of the chain size characteristics. Simulation snapshots in Figures S11 and S12 might help the reader to realize the situation in the simulation system at high particle densities.

Figures 5 and S5 show the variation of chain size with respect to polymer concentration, obtained from simulation data. The color code corresponds to various (fixed) degrees of ionization α. The dashed and solid lines belong to *strong* and *weak* polyelectrolytes, respectively. Note that these systems differ only in the possibility of “reshuffling” of the charges among the polyelectrolyte segments for the weak polyelectrolyte systems. In these cases, there is a narrow distribution of charges among the chains in the system (Figure S20). The systems with lower degree of ionization clearly depict the transition between the two scaling regimes: *R* ∝ *c* ^0^ in the dilute regime^8^ and *R* ∝ *c* ^–1/4^ in the semidilute regime.^17^ Note that the relation *R* ∝ *c* ^–1/4^ was derived for a fully charged chain^40^ while for a neutral chain it is *R* ∝ *c* ^–1/8^. The corresponding dotted lines in Figure 5 serve as an aid to the eye. Our observed dependencies for partly charged chains lie in between. The overlap concentration depends on the degree of ionization as c* ∝ α^–12/7^ (see ref^17^) and therefore the transition between the two above-mentioned regimes lies out of the range of Figure 5 for higher degrees of ionization. In these cases, we do not observe the part, where the chain size does not depend on polymer concentration. We observe only the part where the chain size decreases with concentration, but the scaling exponent is smaller than −0.25. The dependencies for higher degrees of ionization in Figure 5 are “incomplete”, because the simulations for these systems at higher densities are enormously costly. However, the overall scaling and trends are observable also from the currently available data sets. The most important message of Figure 5 is the negligible difference between the chain size of polyelectrolytes with fixed and dynamically adjustable positions of charged sites at comparable conditions. As expected, the chain size increases with the degree of ionization not only in the dilute regime,^13^ but also beyond the overlap concentration.

**Figure 5:**
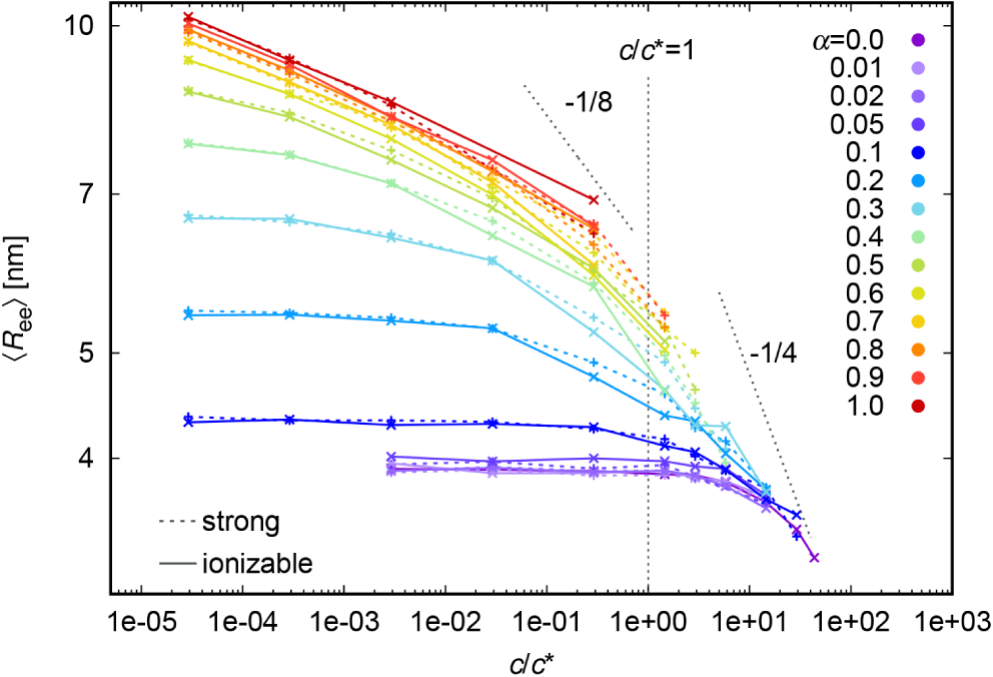
Variation of *R*_ee_ with concentration, with depicted dependency from eq 13.

#### Scaling Relations

The features of scattering curves are related to the conformational characteristics. In particular, the correlation length, ξ, is related to q* as ξ = 2π/q*. Figures 6 and S6 show the relationship between chain size (*R*_ee_ and *R*_g_, respectively) and q*. For very low degrees of ionization (below α = 0.1), the chain size is independent of q*: *R*_ee_ = 4 nm or *R*_g_ = 1.6 nm. The depicted dotted lines and exponents come from a least-squares fitting procedure. Here we need to emphasize, that these particular results are for our coarse-grained simulation model with chain length 50 beads. For higher degrees of ionization we observe other dependencies:

10 $$R_{\mathrm{ee}}\propto \xi^{0.31}\approx \xi^{1/3}$$

in Figure 6 and

11 $$R_{g}\propto \xi^{0.23}$$

in Figure S6. The depicted dotted lines and exponents come from a least-squares fitting procedure. In the semidilute regime,^17^ chain size *R*_ee_ and correlation length are not directly proportional, but related via the blob model as

12 $$R_{\mathrm{ee}}\propto \xi N^{1/2}g^{-1/2}$$

where *N* is the chain length (constant for all simulations) and *g* is the number of monomers in the correlation blob. However, this theoretical blob model uses several approximations, which might not be fulfilled not only in simulations but also in experiments. Theory assumes that the chain lengths are asymptotically limited to infinity, and the chain behaves like a collection of fully independent well-defined correlation blobs. In contrast, the chains in our simulations have a finite length and behave more like single extended objects than a collection of independent blobs. Moreover, the blob model does not take into account local structural effects, such as chain stiffness and counterion condensation. Instead of using eq 12 to compare our results with, we use the following: Combining experimentally obtained dependencies for *R*_g_ ^50^ for α = 1, high concentration and assuming same scaling as for *R*_ee_ we have

13 $$R_{\mathrm{ee}}\propto c^{-1/4}$$

and

14 $$\xi \propto c^{-1/2}$$

Using eq 13 and eq 14, we get the following:

15 $$R_{\mathrm{ee}}\propto \xi^{1/2}$$

which is a stronger dependency than that depicted in Figure 6.

**Figure 6:**
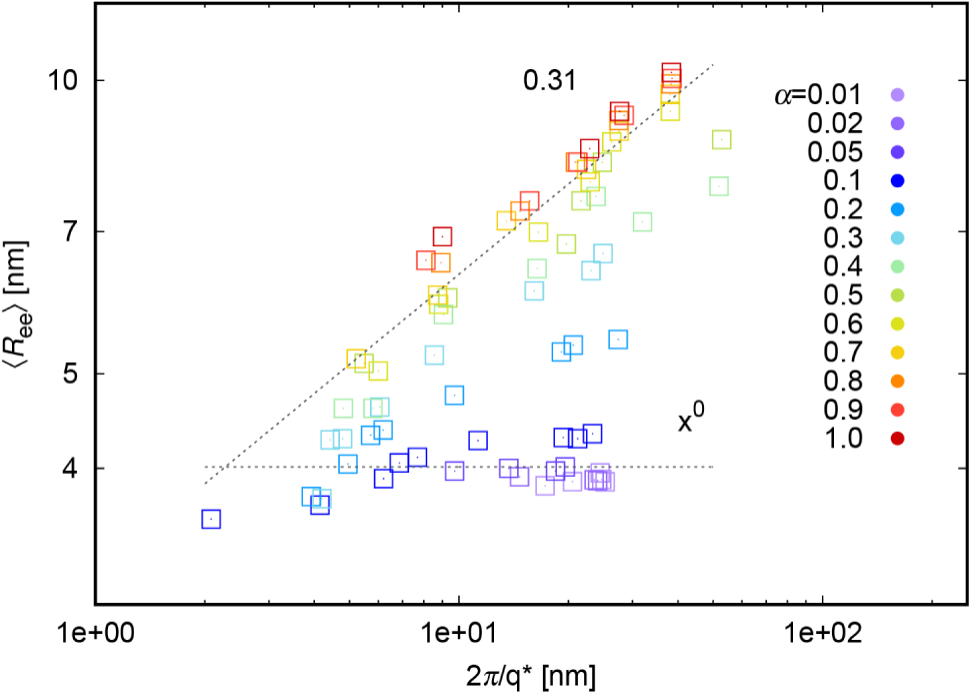
Scaling of *R*_ee_ with q*; with depicted dependency from eq 10, which differs from the one predicted in eq 15.

Figure S4 shows the relation between the chain size (*R*_ee_) and *d*_f_. The relation between *R*_ee_ and *d*_f_ for a neutral chain (eq 9) follows from theory,^40^ which predicts

16 $$R_{\mathrm{ee}}\propto N^{\nu }=N^{1/d_{f}}$$

Indeed, the dependence of *R*_ee_ on *d*_f_ is a constant for systems with low degrees of ionization: *R*_ee_ ∝ 4 nm. However, for more charged chains (α  > 0.3), the connection between *R*_ee_ of a polyelectrolyte chain and its degree of ionization is much more intriguing than it would follow from eq 16. The data follow a universal scaling $R_{\mathrm{ee}}\propto d_{f}^{-2.8}$ , regardless of the particular degrees of ionization. The depicted dotted lines and exponents come from a least-squares fitting procedure. Chain lengths in our simulations are quite limited and values of *d*_f_ are not well-defined so we do not draw any general conclusions from them.

In the semidilute regime,^17^ *R*_ee_ scales with the degree of ionization as

17 $$R_{\mathrm{ee}}\propto \alpha^{1/7}$$

Figure 7 depicts the mutual dependence of *R*_ee_ and α from simulation data for *weak* and *strong* polyelectrolytes. The color code has the meaning of concentration. The depicted black dotted lines and exponents are obtained from a least-squares fitting procedure, while the light dotted lines are guides to the eye. As already noted above, the chain size is independent of the degree of ionization at low degrees of ionization. Above α ≈ 0.1, *R*_ee_ scales with α^0.57^ for lower concentration and α^0.53^ for higher concentrations. This means that in this region, the *R*_ee_ values of our simulation chains correspond to the size of an extended chain formed by electrostatic blobs.^17^ We ascribe the slightly lower scaling coefficient for higher concentration to lower number of reliable data. However, at α ≈ 0.5, the scaling exponents are smaller and we were able to find two more scaling regimes *R*_ee_ ∝ α^2/7^ (simply a transition regime) and *R*_ee_ ∝ α^1/7^ for systems with almost totally charged chains, which is in agreement with theory.^17^ Again, these scaling exponents do not depend on polymer concentration, which means that the ionization-concentration interplay is here quite nicely separable.

**Figure 7:**
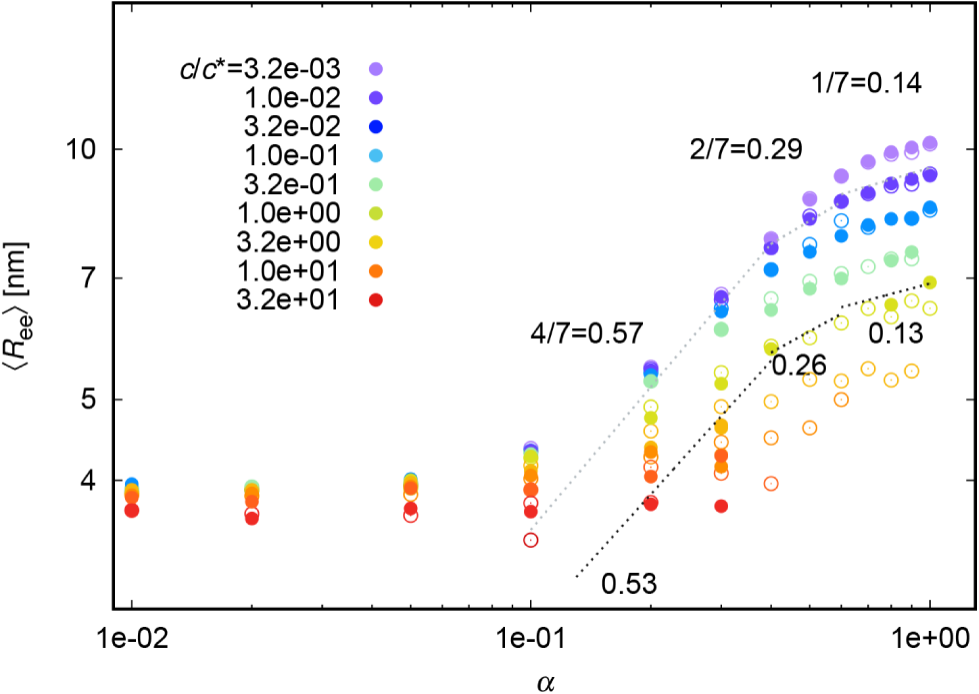
Scaling of *R*_ee_ with α for *weak* (full circles) and *strong* (empty circles) polyelectrolytes; with depicted exponents from eq 17.

#### A Note on Realistic Weak Polyelectrolyte Samples

Unlike in the previous paragraphs, in real experiments with weak polyelectrolytes, we cannot have the degree of ionization fixed and let the only difference between strong and weak polyelectrolytes be the mobility of the charge among the polyelectrolyte beads. Instead, in real weak polyelectrolyte systems, the degree of ionization depends on pH – p*K*_A_. and Figure S7 depict the dependence of the chain size of weak polyelectrolytes on concentration for various pH – p*K*_A_ values. Like in the previous “constant α” case, the chain size monotonously decreases with polymer concentration. At high polymer concentrations, the chain size approaches that of a neutral polymer. This is accompanied by a decrease in the degree of ionization with increasing concentration, ultimately tending toward α = 0 for lower pH – p*K*_A_ values (Figure 9). This reduction in ionization is driven by increasingly unfavorable electrostatic interactions between neighboring like-charged segments. In this regime, the reduction in chain size arises from two synergistic effects: spatial confinement due to crowding, and a decrease in ionization, which both weakens intrachain electrostatic repulsion. At low concentrations, intrachain repulsion dominates; chains are more extended and ionization is higher.^51,52^ In contrast, within the semidilute regime, interchain electrostatic repulsion and excluded volume effects become more prominent.^17,51^ The concurrent decrease in both chain size and ionization with increasing concentration provides strong evidence that interchain interactions are the dominant factor influencing chain conformation in this regime. Therefore, when using simulation results to analyze the concentration dependence of chain size in real weak polyelectrolyte systems, it is important to account for the correlation between degree of ionization and polymer concentration. This coupling introduces uncertainty in the determination of intrinsic chain size.

**Figure 8:**
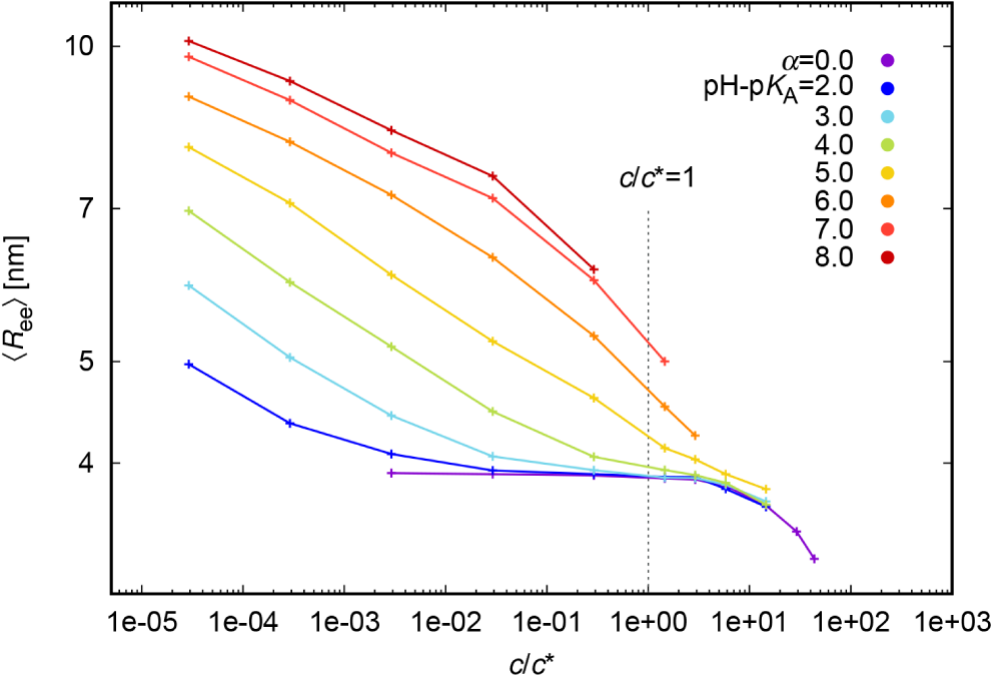
Variation of *R*_ee_ with density for linear weak polyelectrolyte chains.

**Figure 9:**
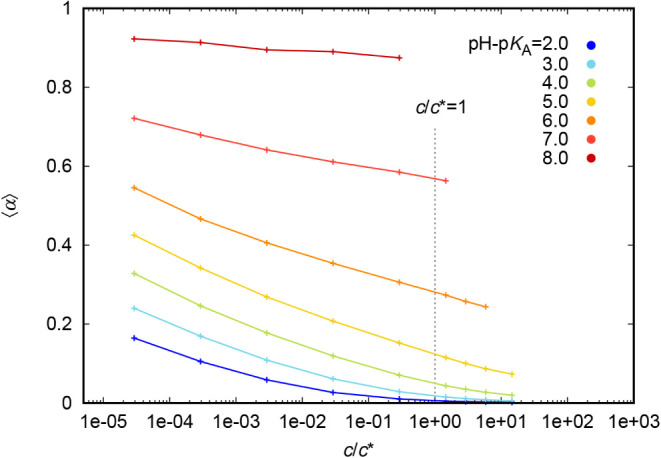
Variation of α with density for linear weak polyelectrolyte chain.

Moreover, Figure 9 reveals an interesting fact that, having pH – p*K*_A_ fixed, the decrease of the degree of ionization with concentration is highest for pH – p*K*_A_ = 5 and pH – p*K*_A_ = 6 (which cover the range around α ≃ 0.6–0.2) than for higher or lower pH – p*K*_A_ values. The systems at pH – p*K*_A_ = 5 and pH – p*K*_A_ = 6 correspond approximately to the condition pH – p*K*_A_app__ = 0, making these systems most responsive to changes in solution conditions. In contrast, especially the system at pH – p*K*_A_ = 8 (in reality almost a strong polyelectrolyte) prefers to stay ionized. This happens despite the many counterions that occur in this system (containing more particles with their own excluded volume).

We can plot these results as titration curves (Figure 10). As expected, the titration curves of polyelectrolyte chains are shifted toward higher pH – p*K*_A_ values with respect to the ideal titration curve^6^ (black dotted). The points in these titration curves are rather scarce because, especially the points at higher degrees of ionization and high concentration, were computationally expensive.

**Figure 10:**
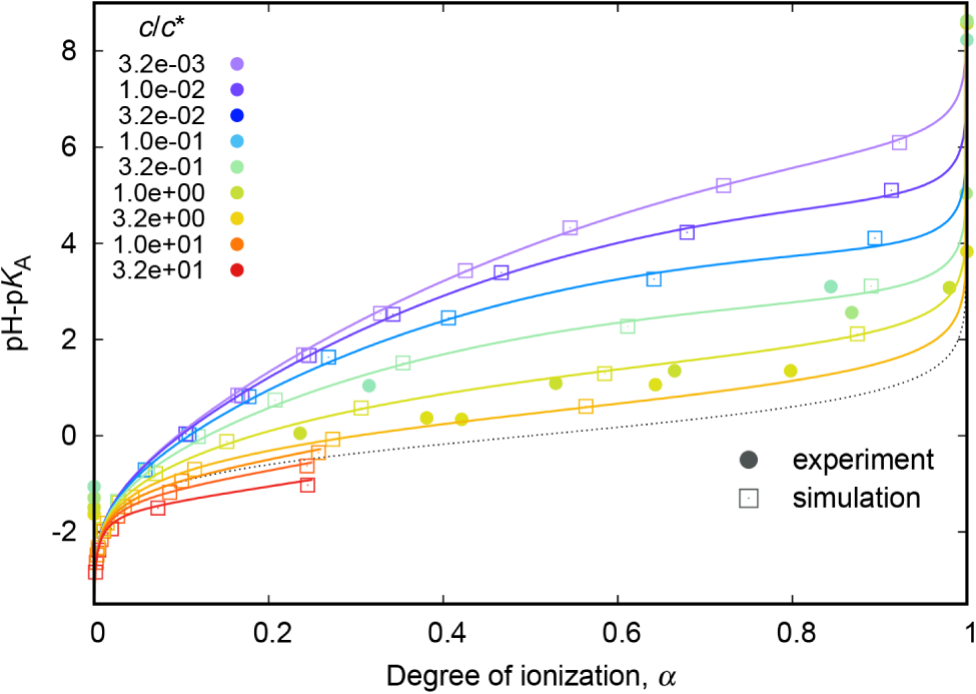
Titration curves for various concentrations.

The points corresponding to higher concentrations appear on the left side of the ideal titration curve. This behavior was already reported^6^ for the titration curves of weak polyelectrolytes in media with low relative permittivity, where charged particles influence each other over longer distances. A similar situation arises at higher concentrations—not due to a lower permittivity, but because the particles are spatially closer, and thus their electrostatic interactions are stronger. We adjusted the relative permittivity values according to eq 7. However, even at *c*/c* ≐ 6, where the titration curve matches the ideal titration curve, the corresponding value is ϵ = 74, which is still relatively close to the value for pure water (ϵ = 80; see Table S2 for used values of ϵ).

We attempted to fit the titration curves from simulations to^53^

18 $$\mathrm{pH}-pK_{A}=k\alpha -\log\frac{1-\alpha }{\alpha }$$

where *k* depends on the electric charge, ion size and permittivity and here we take is as an adjustable parameter. The term *k*α in eq 18 accounts for the known distortion of titration curves of polymeric electrolytes. This equation gives the ideal titration curve for *k* = 0. However, this equation was unable to adequately describe our simulation data, especially at higher degrees of ionization. Therefore, we used the activity coefficient correction in the form of an empirically extended Debye–Hückel approximation (eq 19):

19 $$-\log\gamma =\frac{A\left|z^{+}z^{-}\right|\sqrt{I}}{1+Br\sqrt{I}}$$

where *I* is the ionic strength, *r* is the effective ion diameter in Å and *A* = 0.5085, *B* = 0.3281. The full formula for fitting was then

20 $$\mathrm{pH}-pK_{A}=k\alpha -\log\frac{1-\alpha }{\alpha }+k_{\gamma }\frac{A\left|z^{+}z^{-}\right|\sqrt{I}}{1+Br\sqrt{I}}$$

Even this basic empirical approximation of the activity coefficient completed the shape of the simulation titration curve. The respective fitted parameters are listed in Table S1 and their concentration dependence is graphically displayed in Figure S10. The full lines in Figure 10 are the fitted lines using eq 20 for *c*/c* > 3.2. Data sets for higher *c*/c* values are incomplete and therefore could not be subjected to a reliable fitting procedure. Instead, we used cubic splines to aid the eye in these cases.

We also used cubic splines for all available data sets, and we differentiated the splined curves (Figure S9). The maxima in the differentiated curves correspond to the positions of the inflection points in the titration curves and therefore to the effective pH – p*K*_A_ values.

Figure 10 also contains points from the experiment. Unlike in simulations, we do not know the degree of ionization directly. The full points in Figure 10 depict the dependence of degree of ionization on pH – p*K*_A_, where the pH value was measured and we fixed p*K*_A_ = 4.25.^54^ These points do not come from a real titration; the degree of ionization values were estimated by NMR, from the −COOH shifts. The decrease in the effective p*K*_A_ with increasing concentration was already experimentally observed in ref 55; these titration curves tend to have similar shift in p*K*_A_ as our data, but they are much less distorted (Figure S8). We need to keep in mind that their experimental sample had *M*_w_ = 50 kg/mol, which is about five times less than our sample. The reader might compare the full points from Figure 10 with the full points in Figure S8, where the degree of ionization is calculated from the Henderson–Hasselbalch equation, and the experimental titration curves have different shapes than the titration curves from simulations. The experimental curves in Figure S8 are much less steep at higher pH – p*K*_A_ values, which means that the Henderson–Hasselbalch equation tends to underestimate the real degree of ionization of polyelectrolytes at higher concentrations and at higher pH – p*K*_A_ values, especially when applied with p*K*_A_ value for monomeric acid.

### Study Limitations and Future Perspectives

Like any other simulation study, our research has limited validity, resulting primarily from the limitations of our coarse-grained model. Moreover, using an implicit solvent in highly concentrated systems could further increase the systematic deviation from reality.^28^ We believe that these concerns would be alleviated by using physics-based but more realistic potentials. In the future, we plan to build up our own coarse-grained force field, based on all-atom simulations of semidilute polyelectrolyte-containing systems.

Unfortunately, our simulation series contain fewer points than anticipated, because we did not want to present any results obtained with poor sampling of the conformation space. As already mentioned above, the simulations at higher concentrations and higher degrees of ionization are very computationally costly. In the future, we want to overcome this difficulty by improving our Hamiltonian Monte Carlo technique so that the MD proposal for MC leads to enhanced sampling.

## Conclusions

We studied the conformation and ionization of weak polyelectrolytes in the semidilute regime using SAXS and coarse-grained molecular simulations. We compared the experimental and simulation data in terms of scattering curves. Regarding q*, our simulation data were in agreement with the experiment and therefore we could attempt to draw relevant universal structural information from our simulation data. Regarding *d*_f_, our coarse-grained model of polymer chain with implicit solvent was found inappropriate to provide scattering curves comparable with experiment.

Interestingly, we did not reveal any differences between the morphologies of strong and weak polyelectrolytes at the same degree of ionization in the semidilute regime. However, for real weak polyelectrolytes, the degree of ionization decreases with concentration, and therefore their chain size is smaller. The decrease of the degree of ionization with increasing concentration is most apparent around α ≈ 0.5, but it is much lower at α  >  0.7.

Our data supported the previously predicted scaling relation for polyelectrolytes. We found *R*_ee_ ∝ α^1/7^ for highly charged chains (for α  > 0.5), but we found *R*_ee_ ∝ α^4/7^ for lower degrees of ionization, which corresponds to the predicted scaling for an extended chain formed by electrostatic blobs.

We find that for our simulation setup, $R_{\mathrm{ee}}\propto {\frac{2\pi }{q^{*}}}^{0.31}$ . This relation holds for α > 0.3, i.e., from the point where q* is independent of the degree of ionization. For α < 0.3 we found *R*_ee_ to be constant, independent of the degree of ionization. However, due to limited validity of our simulation setup, we did not use this information to draw relevant conclusions.

## Supplementary Material



## Notes

Characteristics of scattering curves; *R*_g_ from CG simulations; titration curves; simulation snapshots; determination of *c** for experimental sample; permittivity values in simulations; experimental scattering curves; distributions of charges in systems mimicking weak polyelectrolytes (PDF)
