# Supplementary material for: Ionization and Chain Size of Weak Polyelectrolytes in Semidilute Regime
Source: Macromolecules. 2025 Sep 8;58(18):9962–71. doi: 10.1021/acs.macromol.5c00895 (PMC12462241; doi:10.1021/acs.macromol.5c00895)
Supplement: Supplementary file 1 [file ma5c00895_si_001.pdf]

Supporting Information
Ionization and chain size of weak polyelectrolytes in semidilute regime

Lucie Nová,[1,*] Miroslav Štěpánek,[1] Iryna Morozova,[1] Zdeněk Tošner,[1] and Filip Uhlík[1]

[1]*Department of Physical and Macromolecular Chemistry, Faculty of Science, Charles University, Hlavova 8, 128 00 Prague, Czech Republic*

CONTENTS

I. Characterisctics of scattering curves 3

II. Rg from CG simulations 5

III. Titration curves 7

IV. Simulation snapshots 9

V. Determination of c^* for experimental sample from DLS 11

VI. Determination of c^* for experimental sample from DOSY NMR 13

VII. Dielectric permittivity values for particular $c/c*$ 15

VIII. Experimental SAXS curves 15

IX. Distributions of charges in systems mimicking weak polyelectrolytes 17

References 18

* Lucie.nova@natur.cuni.cz

This page is intentionally left blank.

I. CHARACTERISCTICS OF SCATTERING CURVES

Similar figures in the main text are in the representation $c/c*$, which sometimes disables direct comparison of our data with previous experimental data from literature (depicted as black crosses).

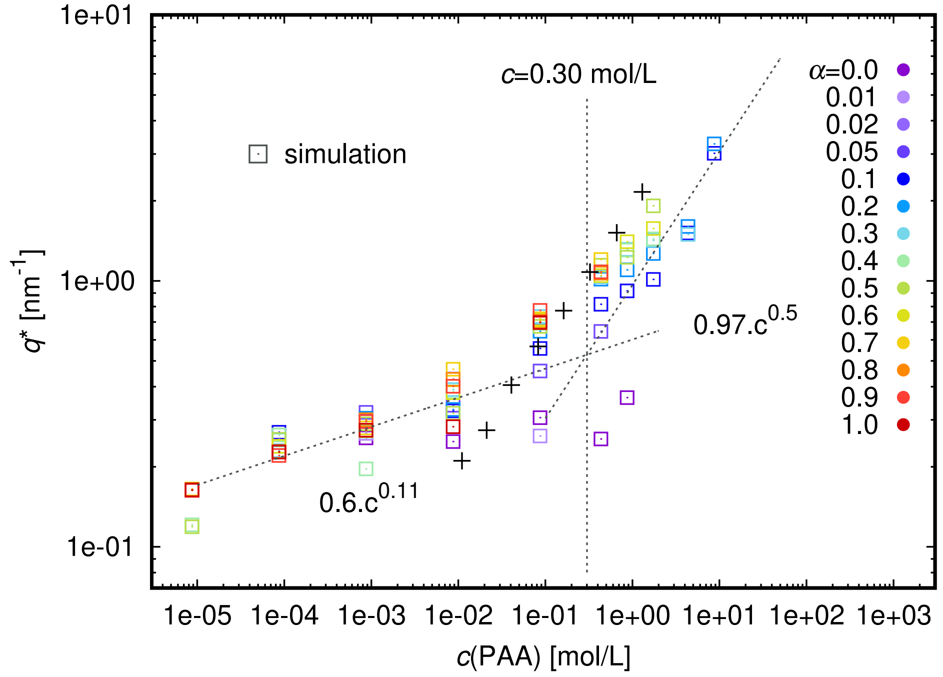

FIG. S1: Variation of $q*$ with PAA concentration with depicted determination of c^*. Black crosses are experimental data from [1].

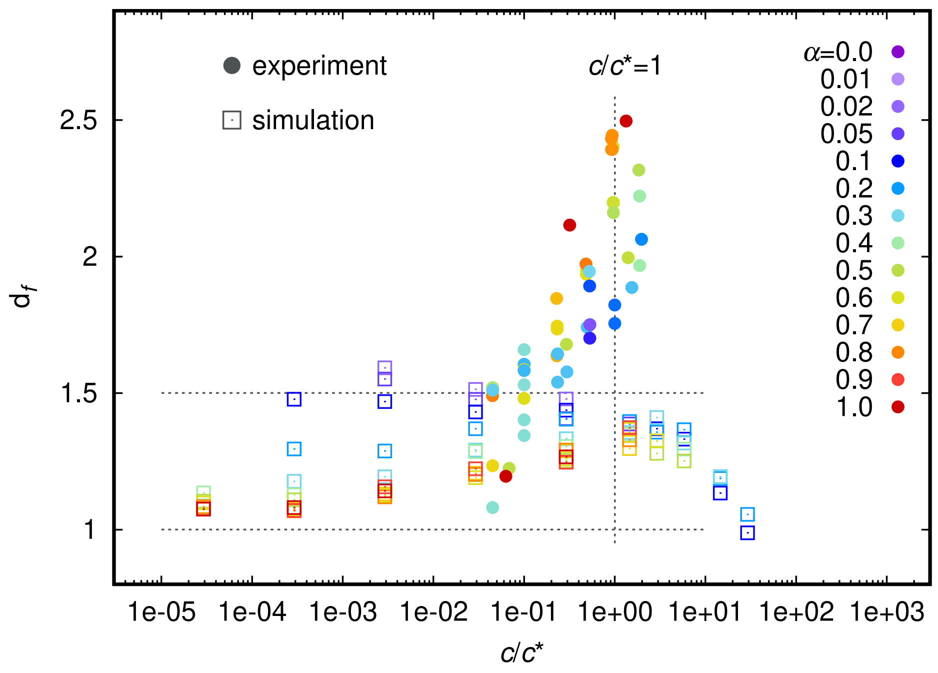

FIG. S2: Variation of d_f with PAA concentration.

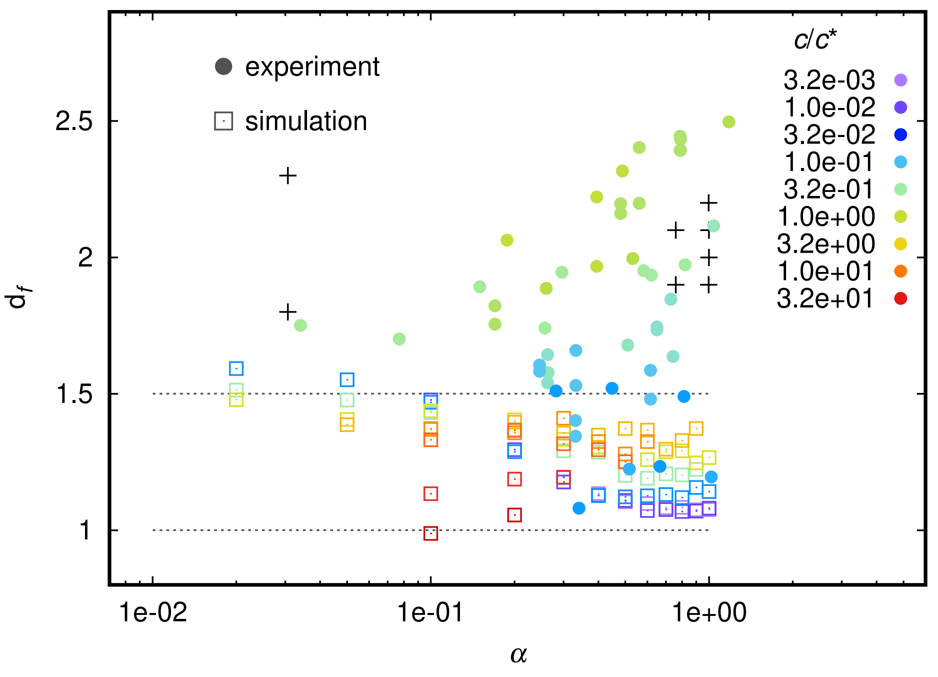

FIG. S3: Variation of d_f with α. Black crosses are experimental data from [2].

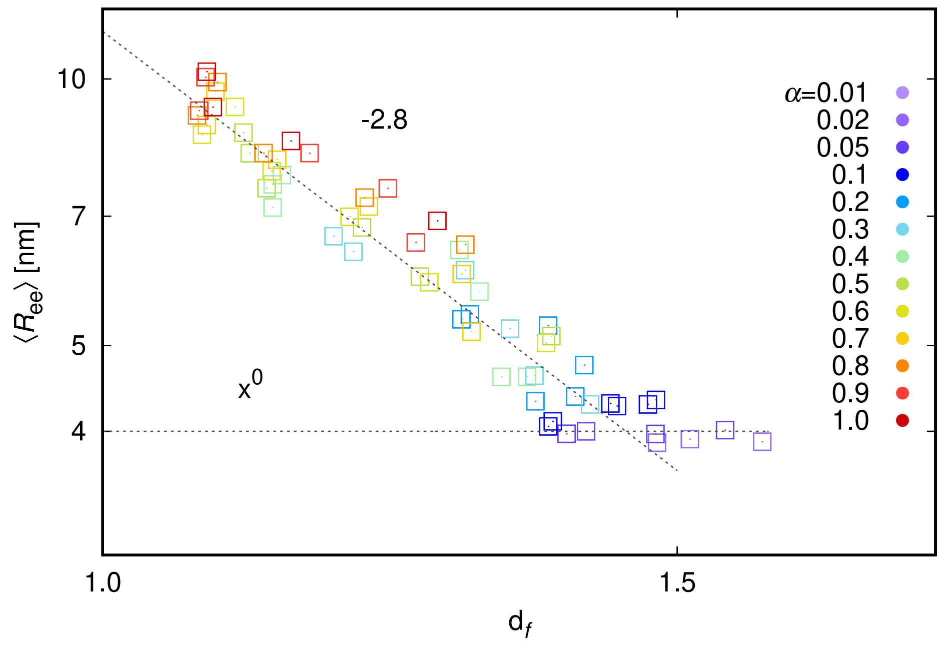

FIG. S4: Scaling of R_{ee} with d_f

II. Rg FROM CG SIMULATIONS

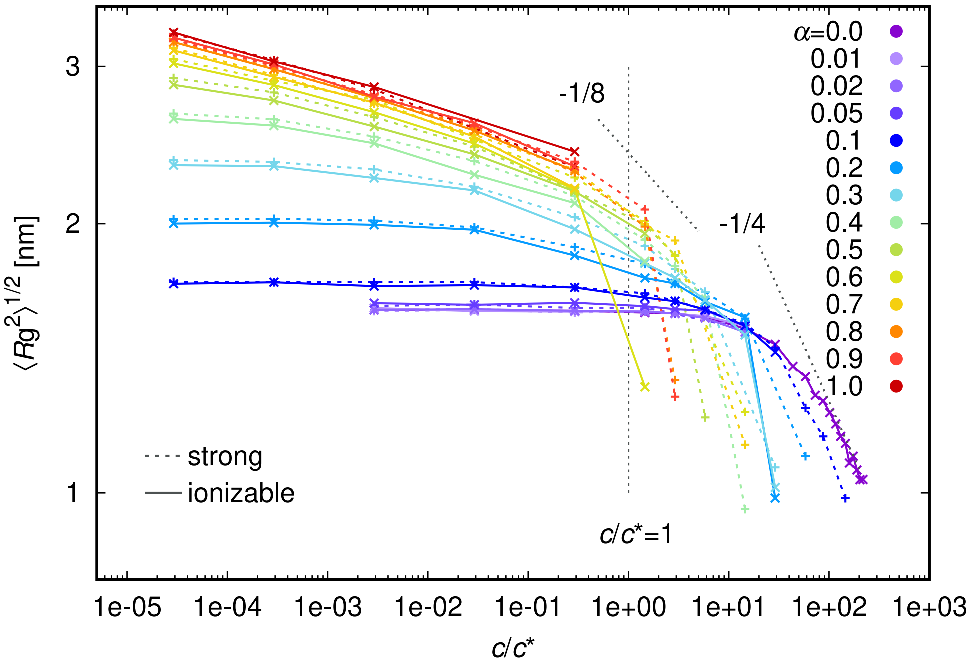

FIG. S5: Variation of Rg with concentration.

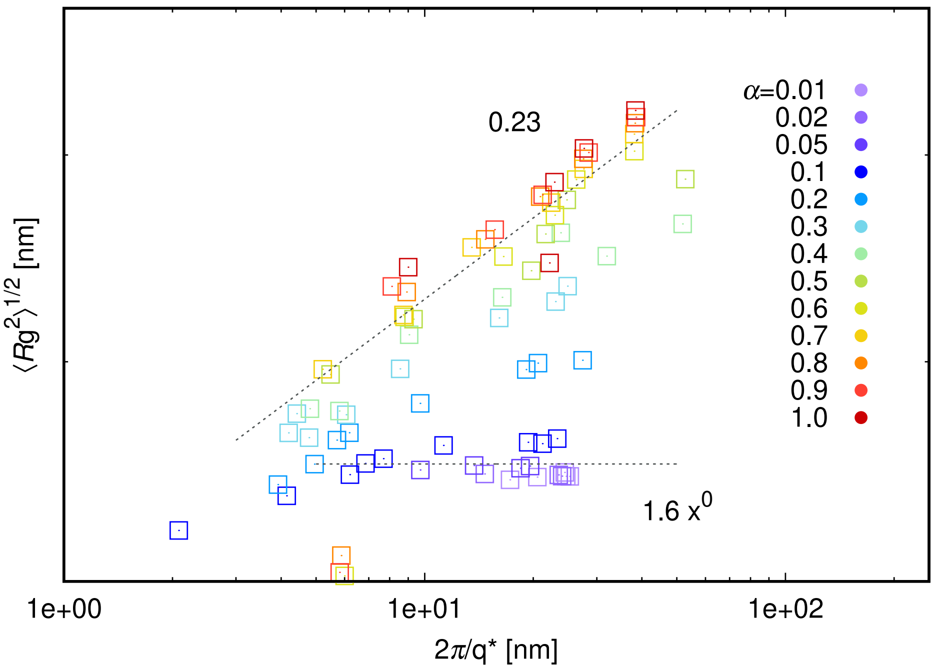

FIG. S6: Scaling of Rg with $q*$, with depicetd dependency from Eq. 11.

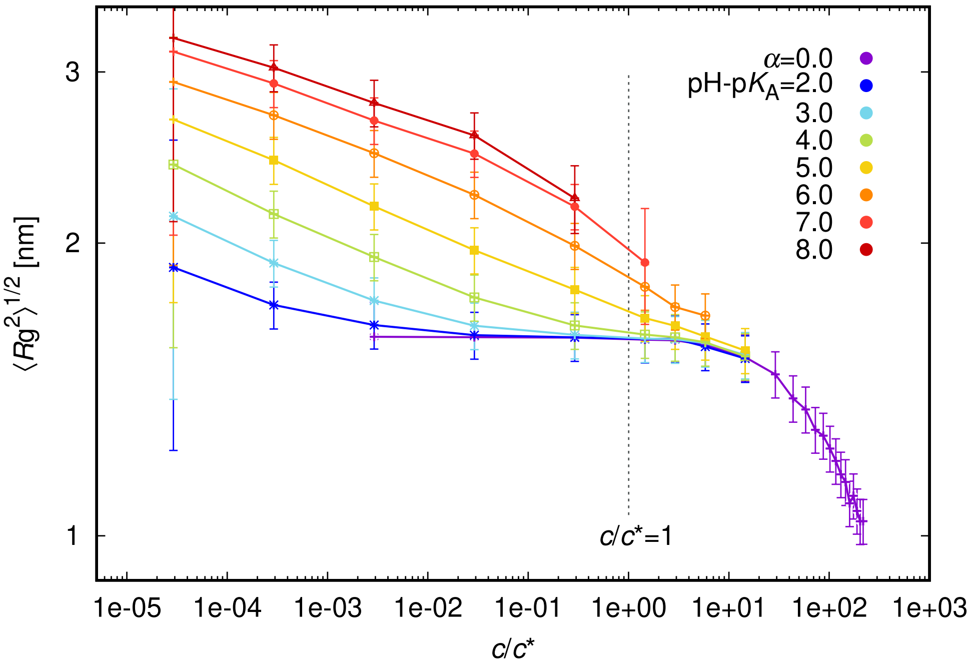

FIG. S7: Variation of Rg with density for linear weak polyelectrolyte chains

III. TITRATION CURVES

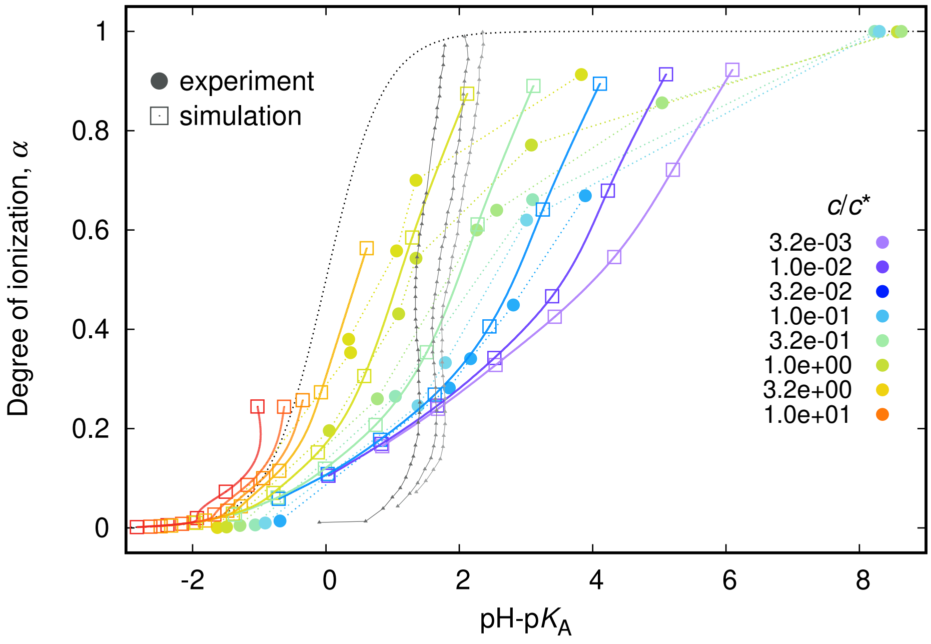

FIG. S8: Titration curves for various concentrations. The gray points are taken from [3]

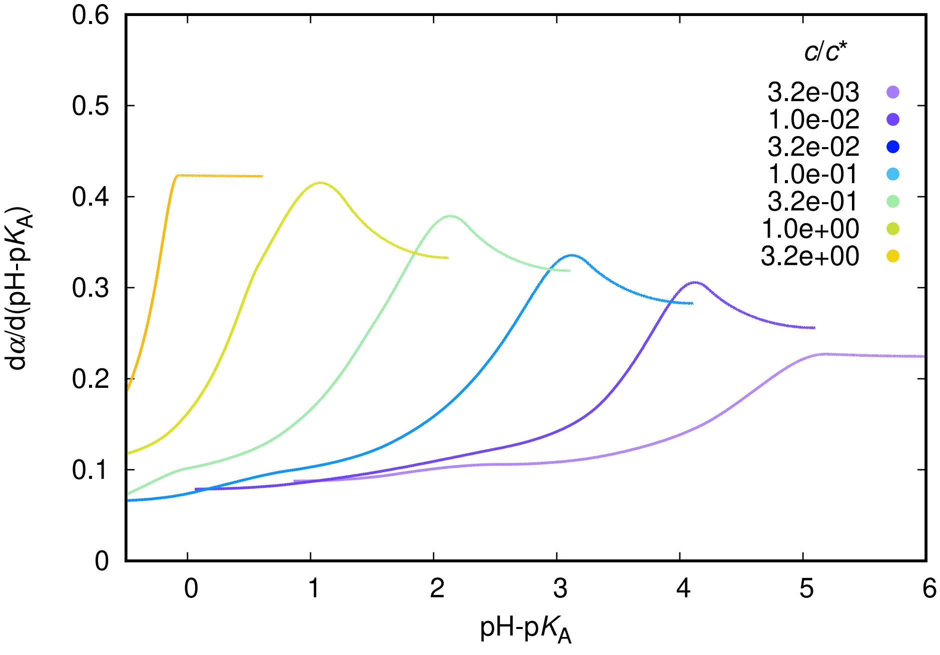

FIG. S9: Derivative of titration curves with applied cubic spline

The simulation titration curves were fitted to the formula

$$\mathrm{pH} - \mathrm{p}K_A = k \cdot \alpha - \log\frac{1-\alpha}{\alpha} + k_\gamma \frac{A|z^+z^-|\sqrt{I}}{1 + B\cdot r\cdot\sqrt{I}} \tag{S1}$$

where I is the ionic strength, $r = 3\mathring{A}$ is the effective hydrogen ion diameter and $A = 0.5085$, $B = 0.3281$. k is the parameter which depends on the electric charge, ion size and permittivity and here we take is as free fitting parameter. We did not distinguish between individual activity coefficients for the polymer anions and hydrogen cations on purpose. Instead, we took into account the one total activity coefficient for the ionization reaction at given conditions. The difference between the individual activity coefficients and the total activity coefficient was accounted using the free fitting parameter k_γ. The fitted parameters are listed below.

c/c^*	k	k_γ
3.2×10^{-3}	10.83(2)	-15.42(6)
1.0×10^{-2}	10.49(2)	-5.83(2)
3.2×10^{-2}	9.26(3)	-1.97(1)
1.0×10^{-1}	7.42(3)	-0.672(5)
3.2×10^{-1}	4.93(2)	-0.242(2)
1.0	2.42(3)	-0.093(2)
3.2	-0.7(1)	0.105(8)

TABLE S1: Fitted parameters to eq. 11

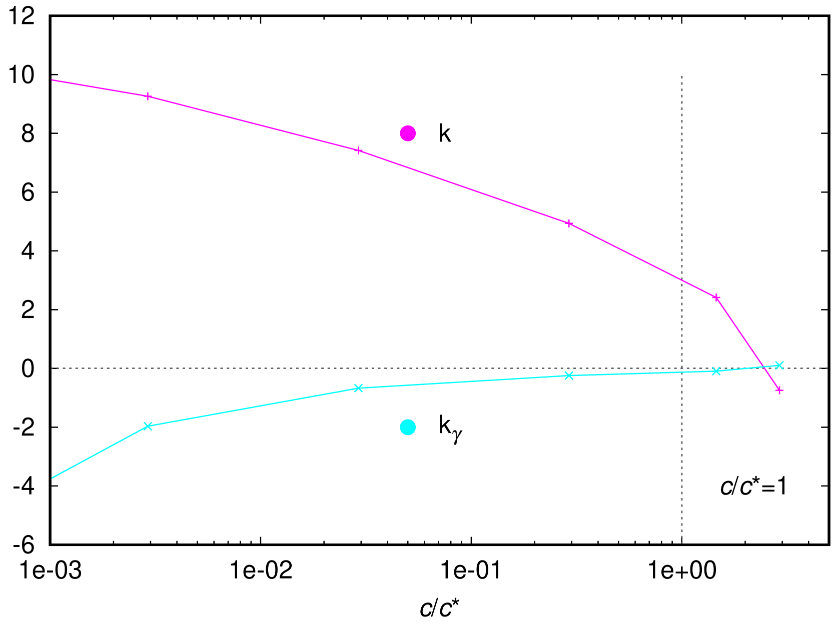

FIG. S10: Concentration dependence of the fitted k and k_γ parameters.

IV. SIMULATION SNAPSHOTS

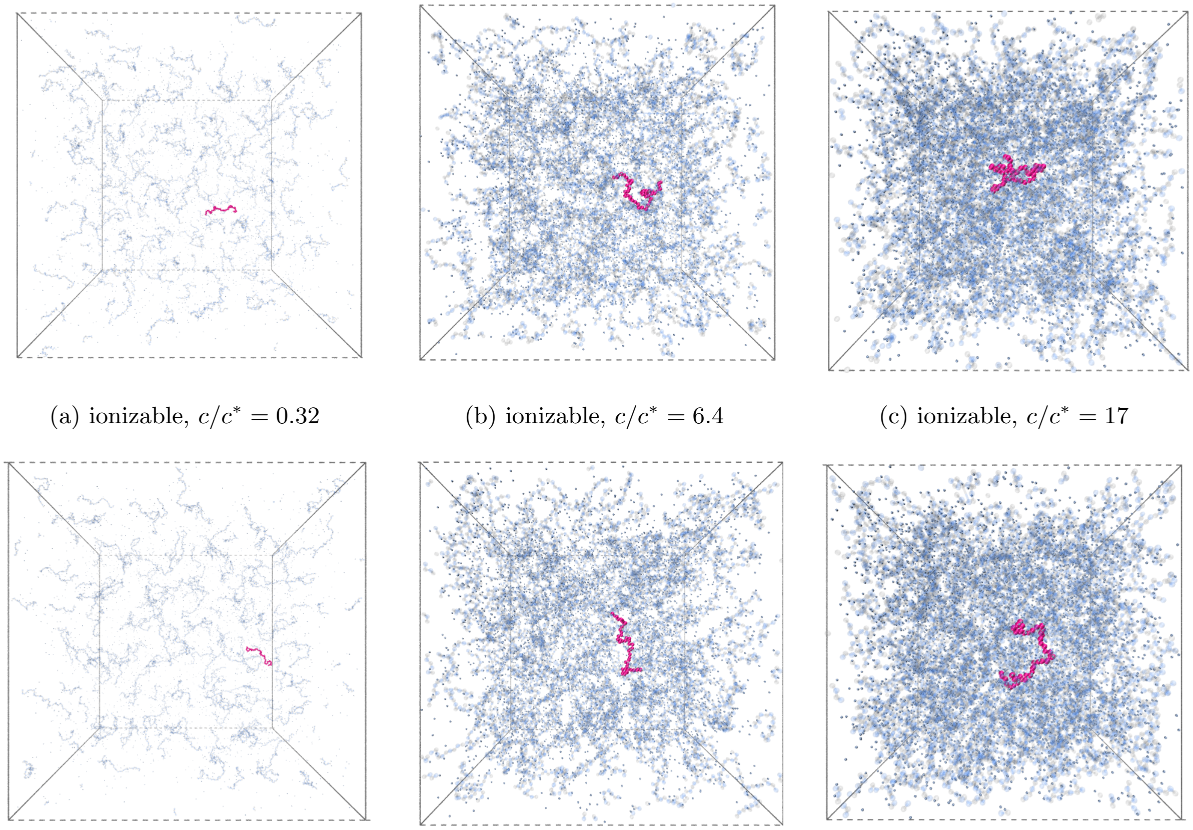

(a) ionizable, $c/c^* = 0.32$ (b) ionizable, $c/c^* = 6.4$ (c) ionizable, $c/c^* = 17$

(d) strong, $c/c^* = 0.32$ (e) strong, $c/c^* = 6.4$ (f) strong, $c/c^* = 17$

FIG. S11: Simulation snapshots of polyelectrolyte chains at various concentrations and at the degree of ionization $\alpha = 0.5$. One chain is highlighted to aid the eye.

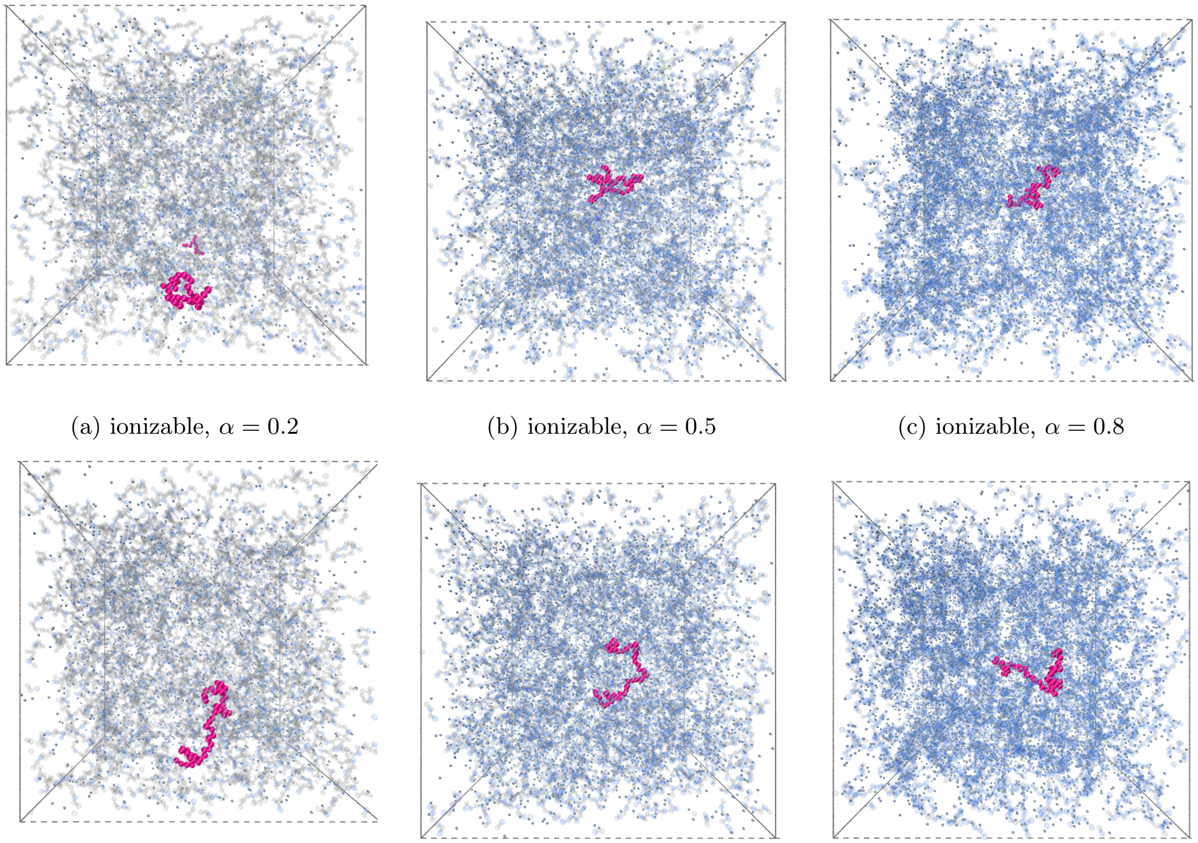

(a) ionizable, $\alpha = 0.2$ (b) ionizable, $\alpha = 0.5$ (c) ionizable, $\alpha = 0.8$

(d) strong, $\alpha = 0.2$ (e) strong, $\alpha = 0.5$ (f) strong, $\alpha = 0.8$

FIG. S12: Simulation snapshots of polyelectrolyte chains at various degrees of ionization, α and $c/c^* = 17$. One chain is highlighted to aid the eye.

V. DETERMINATION OF c^* FOR EXPERIMENTAL SAMPLE FROM DLS

In order to estimate the overlap concentration, we employed dynamic light scattering (DLS) using the fact that after concentration fluctuations in the solution become suppressed with increasing concentration after reaching overlap concentration. We used only non-neutralized samples for this purpose.

The employed light scattering setup (ALV, Langen, Germany) consisted of a 22 mW He-Ne laser, operating at the wavelength $\lambda = 660$ nm, an ALV CGS/8F goniometer, an ALV High QE APD detector, and an ALV 5004 multibit, multitau autocorrelator. The measurements were performed at constant temperature 25°C. The scattering angle θ in the range from 40° to 150°. The measured scattering intensities were converted to Rayleigh ratios using the calibration by toluene standard [4, 5].

DLS measurements were evaluated by fitting the measured normalized time autocorrelation function of the scattered light intensity [4, 5]. In most cases we observed two relaxation modes- the 'fast mode' and the 'slow mode'. From this point of view, all solutions were in the semidilute regime, because the 'slow mode' appears as soon as the solution becomes semidilute [6].

We plotted the angular dependence of the respective relaxation times (Fig. S13) and inspected their scaling via fitting them to power law. The fitted curves and the corresponding coefficients for the fast mode are not depicted,

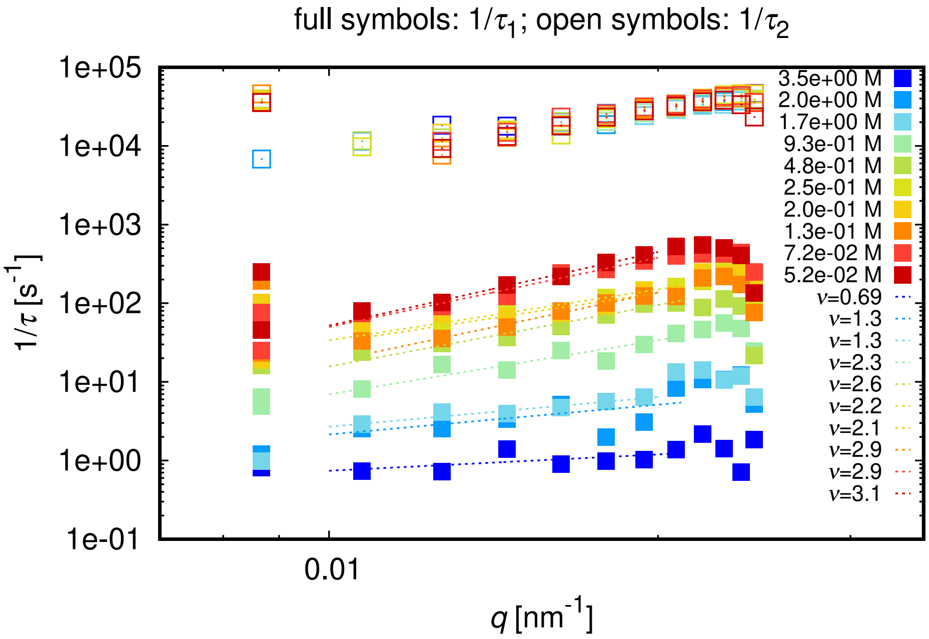

FIG. S13: Angular dependence of relaxation times for slow and fast modes.

for the sake of clarity.

Fig. S14 shows scaling exponents, ν_s and ν_f for the relaxation rates of the slow and fast mode. A similar dependence was studied for semidilute solutions of a neutral polymer (polystyrene) [6], showing that while the fast mode corresponding to the motion of polymer blob has diffusive character ($\nu_f = 2$) over the entire examined concentration range, the ν_s decreases with concentration from 3 to 0 because of different nature of interchain interactions at different concentrations. The entanglement concentration can be estimated as the value at which $\nu_f = \nu_s$. In the case of PAA, ν_f is slightly higher (2.5), most likely because of electrostatic interactions between the blobs, however, using the same assumptions as in [6], we can estimate the overlap concentration as $c^* = 0.45$ mol/L.

c^* value divides Fig. S14 to two concentration regimes, where the lower concentrations correspond to a semidilute, non-entangled regime, while the higher concentrations correspond to a semidilute, entangled regime. The difference between the semidilute, non-entangled and semidilute, entangled regimes also corresponds to the difference in scaling regimes in Fig. 2 in the main text.

Note that, for polyelectrolytes, the overlap concentration depends on the charge on the chain. We are fully aware of this fact and despite it, we used this experimentally determined c^* in the figures in the main text for all degrees of neutralization to depict the dependencies of relevant quantities on the degree of ionization of PAA chains.

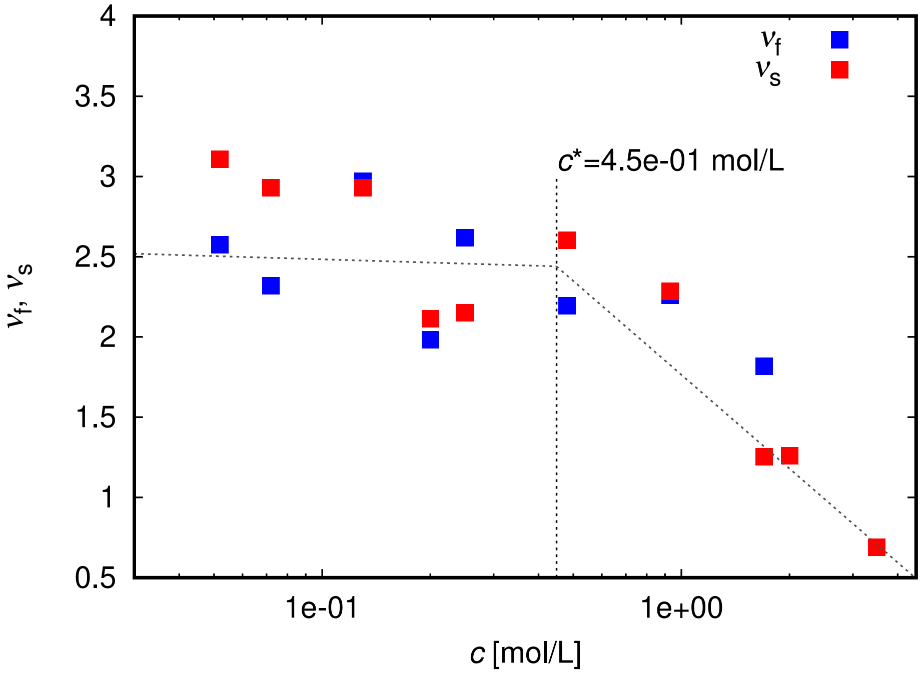

FIG. S14: Concentration dependence of exponents from Fig. S13 for slow and fast modes.

VI. DETERMINATION OF c^* FOR EXPERIMENTAL SAMPLE FROM DOSY NMR

Because the determination of c^* from DLS relies on the slow mode, which is still not generally accepted to be fully understood, we re-estimated the c^* using DOSY NMR.

The samples of various polymer concentrations and degrees of neutralization were measured using a Bruker Avance III spectrometer operating at 600 MHz [7].

We used the fact that the overlap concentration is a concept related to the diffusion behavior in polymers in solutions. Integral intensity of the CH_2 peak at around 2.4 ppm (empty points) and around 1.5 ppm (full points) were evaluated as a function of gradient strength. In order to obtain the self-diffusion coefficient, we fitted our DOSY data to the Stejskal-Tanner equation. The usage of the Stejskal-Tanner equation in its most common form, with only one diffusion coefficient, failed to describe the experimental data. But the data were nicely described using the of sum of two exponentials with two respective diffusion coefficients.

$$I = a_1 \exp[-\gamma^2 g^2 \delta^2 (\Delta - \delta/3) D_1] + a_2 \exp[-\gamma^2 g^2 \delta^2 (\Delta - \delta/3) D_2] \quad \text{(S2)}$$

where γ is the gyromagnetic ratio, g is the gradient strength, δ is the gradient pulse duration, Δ is the diffusion delay and D is the self-diffusion coefficient.

Total signal decay is given as

$$D = a_1 \cdot D_1 + a_2 \cdot D_2 \quad \text{(S3)}$$

where $a_1 + a_2 = 100\%$

We plotted the concentration dependence of these obtained diffusion coefficients, D_1 and D_2, and of their relative percentage incidence, a_1 and a_2, and of the total diffusion coefficient, D, and we found two distinct regimes, which cross at the concentration $c^* \simeq 0.4$mol/L. The c^* values from DOSY NMR agrees with c^* from DLS. For the sake of simplicty, and similarly to the DLS experiment above, we included only the non-neutralized samples into this treatment. More complex study of these phenomena will follow [7].

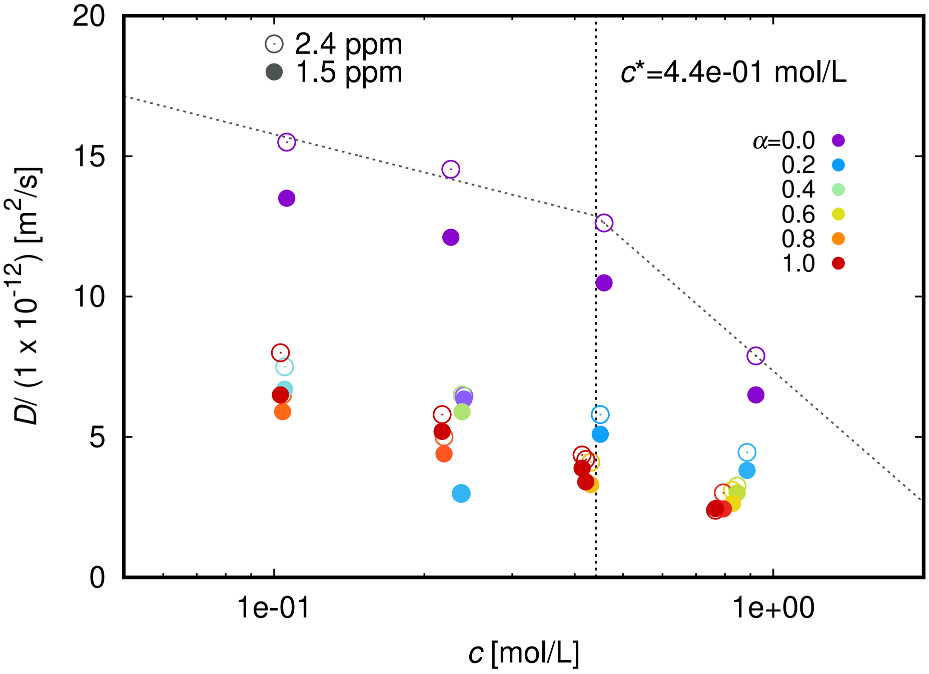

FIG. S15: Concentration dependence of total diffusion coefficients D.

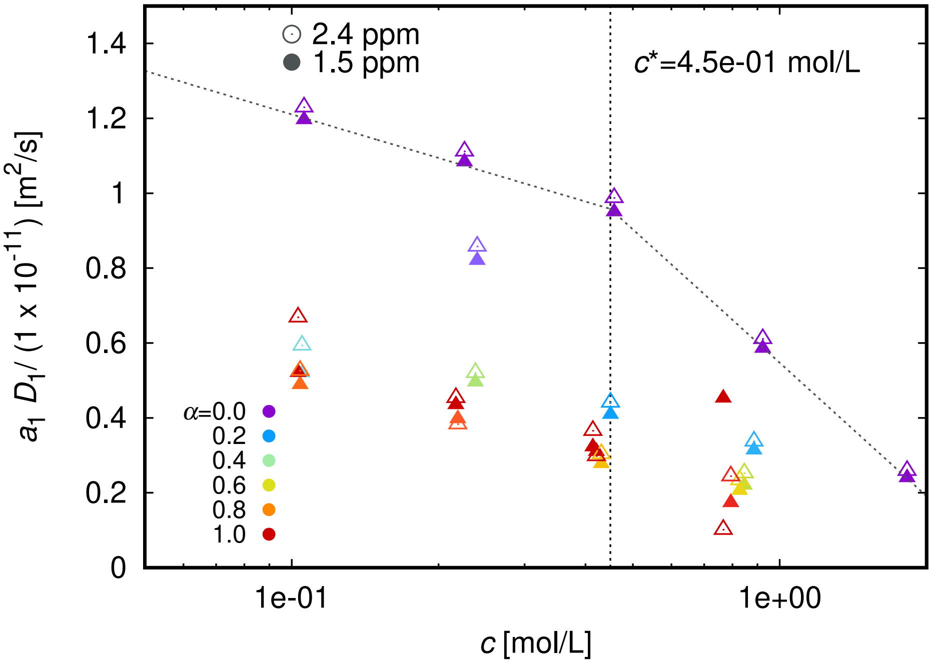

FIG. S16: Concentration dependence of diffusion coefficients D_1.

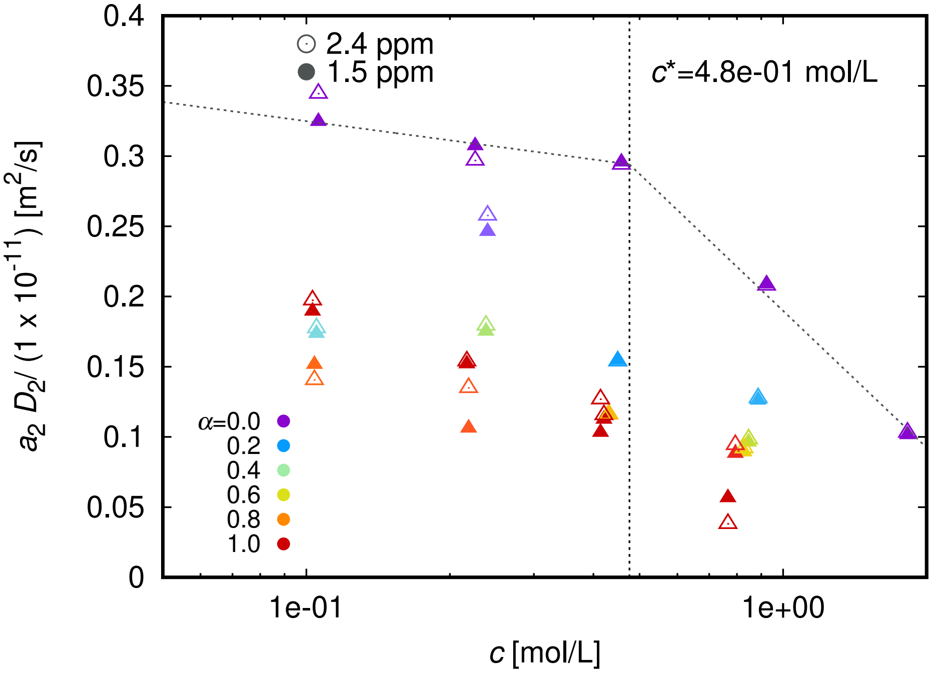

FIG. S17: Concentration dependence of diffusion coefficients D_2.

VII. DIELECTRIC PERMITTIVITY VALUES FOR PARTICULAR $c/c*$

c/c^*	Φ_{water}	ε_r
3.1×10^{-3}	0.99999	80
3.1×10^{-2}	0.99993	80
3.1×10^{-1}	0.99926	80
1.6	0.99630	78
3.1	0.99260	77
6.2	0.98520	74
15	0.96299	66

TABLE S2: Values of relative permittivity for particular volume fractions of water.

VIII. EXPERIMENTAL SAXS CURVES

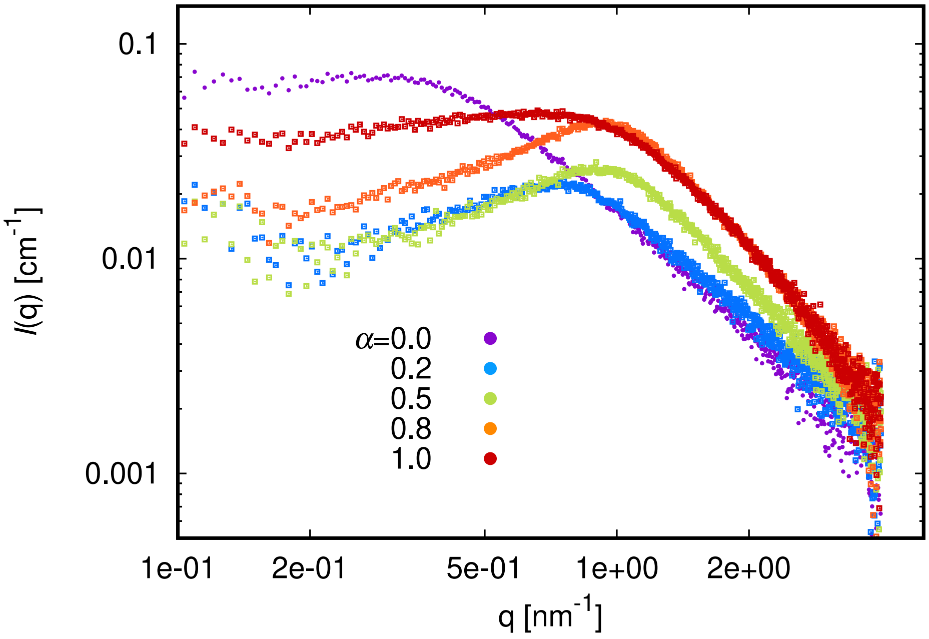

FIG. S18: Experimental SAXS curves for various α at $c/c^* = 1$.

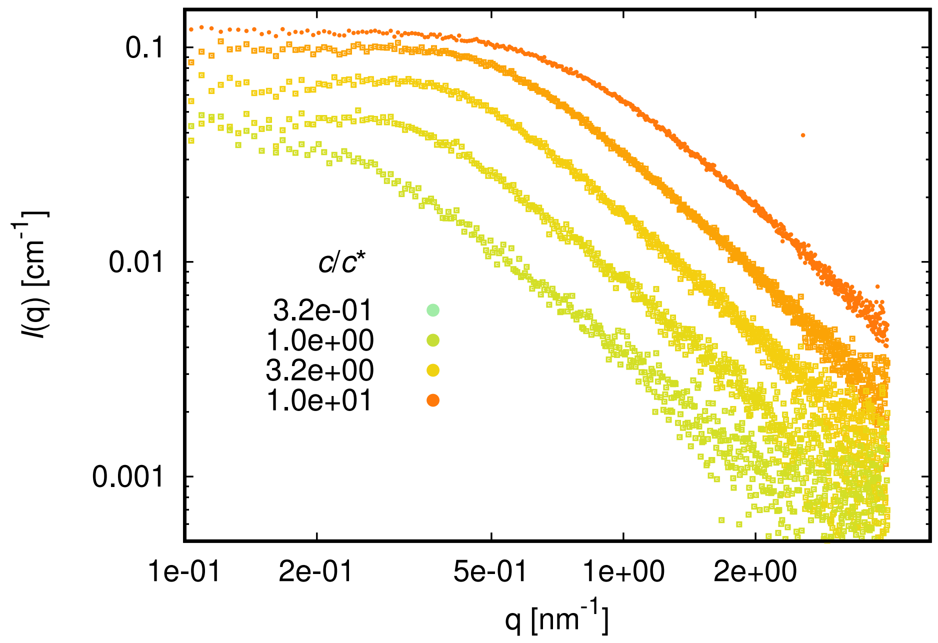

(a) $\alpha = 0$

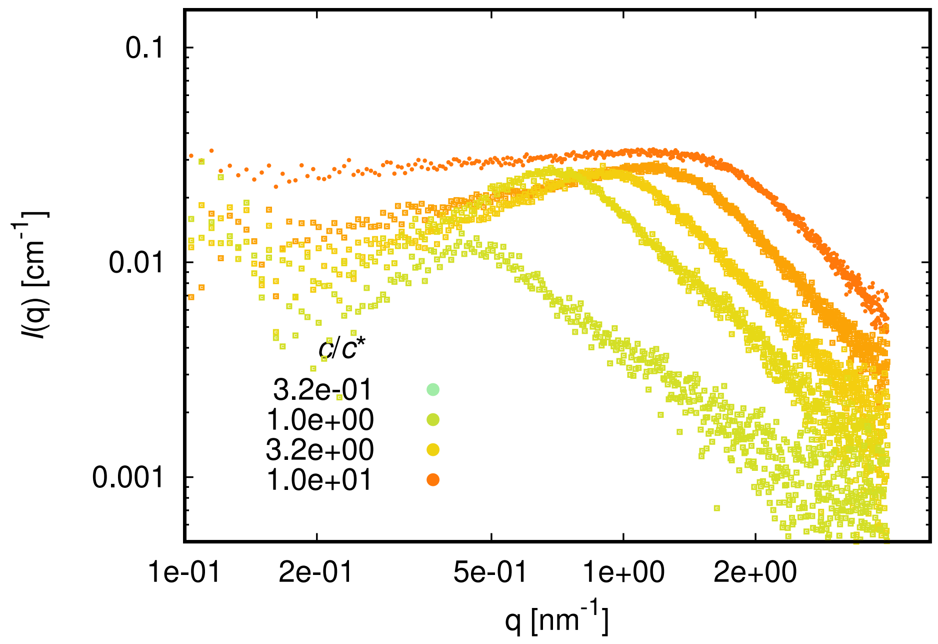

(b) $\alpha = 0.4$

FIG. S19: Experimental SAXS curves for various concentrations at $\alpha = 0$ and $\alpha = 0.4$.

IX. DISTRIBUTIONS OF CHARGES IN SYSTEMS MIMICKING WEAK POLYELECTROLYTES

Below, we show sample distributions of degrees of ionization, α. Assuming normal distribution, the respective σ values are in the range from 0.036 to 0.040.

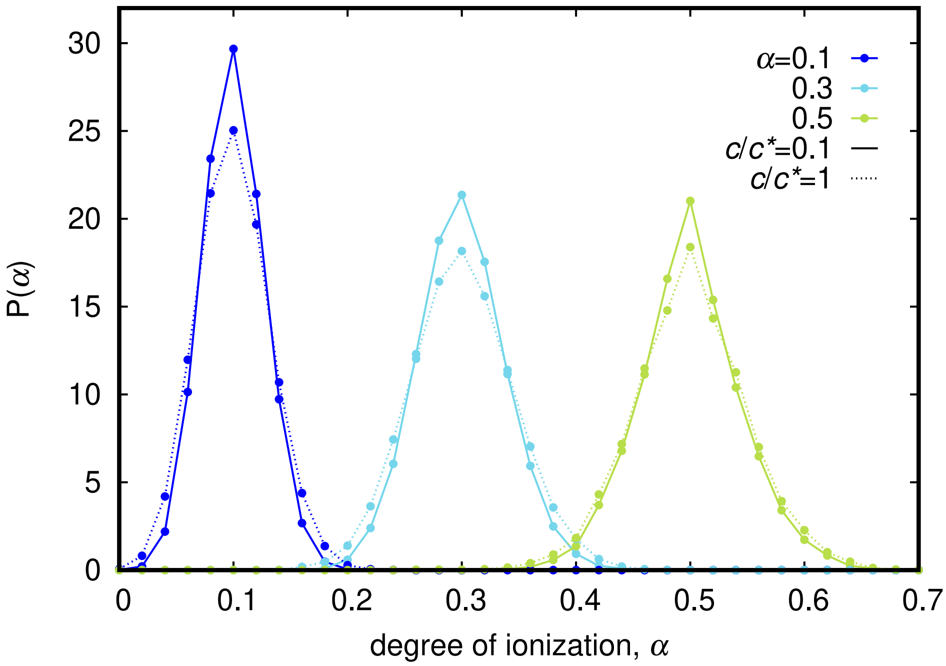

FIG. S20: Distributions of charges among chains in the systems with 'shuffling' reaction.

[1] Combet, Jérôme, EPJ Web of Conferences **188**, 03001 (2018).
[2] K. Alba, R. J. Bingham, and V. Kontogiorgos, Biopolymers **107**, e23016 (2017), https://onlinelibrary.wiley.com/doi/pdf/10.1002/bip.23016.
[3] C. Heitz, M. Rawiso, and J. François, Polymer **40**, 1637 (1999).
[4] A. Fanova, I. Davidovich, Y. Talmon, A. Skandalis, S. Pispas, and M. Štěpánek, ACS Applied Polymer Materials **3**, 1956 (2021).
[5] M. Uchman, J. Hajduová, E. Vlassi, S. Pispas, M.-S. Appavou, and M. Štěpánek, European Polymer Journal **73**, 212 (2015).
[6] J. Li, W. Li, H. Huo, S. Luo, and C. Wu, Macromolecules **41**, 901 (2008).
[7] I. Morozova, Bachelor thesis (2025).
